# MEIGO: an open-source software suite based on metaheuristics for global optimization in systems biology and bioinformatics

**DOI:** 10.1186/1471-2105-15-136

**Published:** 2014-05-10

**Authors:** Jose A Egea, David Henriques, Thomas Cokelaer, Alejandro F Villaverde, Aidan MacNamara, Diana-Patricia Danciu, Julio R Banga, Julio Saez-Rodriguez

**Affiliations:** 1 Department of Applied Mathematics and Statistics, Universidad Politécnica de Cartagena, 30202 Cartagena, Spain; 2 (Bio)Process Engineering Group, Spanish National Research Council, IIM-CSIC, 36208 Vigo, Spain; 3 European Molecular Biology Laboratory, European Bioinformatics Institute, Wellcome Trust Genome Campus, Cambridge CB10 1SD, UK

## Abstract

**Background:**

Optimization is the key to solving many problems in computational biology. Global optimization methods, which provide a robust methodology, and metaheuristics in particular have proven to be the most efficient methods for many applications. Despite their utility, there is a limited availability of metaheuristic tools.

**Results:**

We present MEIGO, an R and Matlab optimization toolbox (also available in Python via a wrapper of the R version), that implements metaheuristics capable of solving diverse problems arising in systems biology and bioinformatics. The toolbox includes the enhanced scatter search method (eSS) for continuous nonlinear programming (cNLP) and mixed-integer programming (MINLP) problems, and variable neighborhood search (VNS) for Integer Programming (IP) problems. Additionally, the R version includes BayesFit for parameter estimation by Bayesian inference. The eSS and VNS methods can be run on a single-thread or in parallel using a cooperative strategy. The code is supplied under GPLv3 and is available at http://www.iim.csic.es/~gingproc/meigo.html. Documentation and examples are included. The R package has been submitted to BioConductor. We evaluate MEIGO against optimization benchmarks, and illustrate its applicability to a series of case studies in bioinformatics and systems biology where it outperforms other state-of-the-art methods.

**Conclusions:**

MEIGO provides a free, open-source platform for optimization that can be applied to multiple domains of systems biology and bioinformatics. It includes efficient state of the art metaheuristics, and its open and modular structure allows the addition of further methods.

## Background

Mathematical optimization plays a key role in systematic decision making processes, and is used virtually in all areas of science and technology where problems can be stated as finding the best among a set of feasible solutions. In bioinformatics and systems biology, there has been a plethora of successful applications of optimization during the last two decades (see reviews in [1-5]). Many problems in computational biology can be formulated as IP problems, such as sequence alignment, genome rearrangement and protein structure prediction problems [1,3], or the design of synthetic biological networks [6]. Deterministic and stochastic/heuristic methods have been extensively applied to optimization problems in the area of machine learning [2]. In addition to combinatorial optimization, other important classes of optimization problems that have been extensively considered, especially in systems biology, are cNLP and mixed-integer dynamic optimization. Such problems arise in parameter estimation and optimal experimental design [5,7].

A number of authors have stressed the need to use suitable global optimization methods due to the non-convex (multimodal) nature of many of these problems [4,8,9]. Roughly speaking, global optimization methods can be classified into exact and stochastic approaches. Exact methods can guarantee convergence to global optimality, but the associated computational effort is usually prohibitive for realistic applications. In contrast, stochastic methods are often able to locate the vicinity of the global solution in reasonable computation times, but without guarantees of convergence. Metaheuristics (i.e. guided heuristics) are a particular class of stochastic methods that have been shown to perform very well in a broad range of applications [5].

Motivated by this, we developed the software suite MEIGO (MEtaheuristics for systems biology and bIoinformatics Global Optimization) which provides state of the art metaheuristics (eSS and VNS) in open-source R (here with the addition of the Bayesian inference method BayesFit) and Matlab versions (it is also available in Python via a wrapper for the R version). MEIGO covers the most important classes of problems, namely (i) problems with real-valued (cNLPs) and mixed-integer decision variables (MINLPs), and (ii) problems with integer and binary decision variables (IPs). Furthermore, MEIGO allows the user to apply parallel computation using cooperative strategies [10]. MEIGO can optimize arbitrary objective functions that are handled as black-boxes. Thus, it is applicable to optimize complex systems that may involve solving inner problems (e.g. simulations or even other optimization problems) to obtain explicit values for the objective function and/or the possible constraints. For example, CellNOpt [11], SBToolbox [12], AMIGO [13] and Potterswheel [14] use eSS for dynamic model calibration. Some recent successful applications of eSS in the field of systems biology can be found in [15-26]. It has also been shown that eSS outperformed the various optimization methods available in the Systems Biology Toolbox [27].

## Methods

### Enhanced Scatter Search (eSS)

Scatter search [28] is a population-based metaheuristic which can be classified as an evolutionary optimization method. In contrast with other popular population-based metaheuristics like, for example, genetic algorithms, the population size, *N*, in scatter search is small, and the combinations among its members are performed systematically, rather than randomly. The current population is commonly named the “Reference Set” (RefSet). The *improvement method*, which consists of a local search to increase the convergence to optimal solutions, can be applied with more or less frequency to the members of this RefSet. A set of improvements has been implemented in the enhanced scatter search method. Among the most remarkable changes, we can mention the replacement method. Unlike in the original scatter search scheme, which uses a *μ*+*λ* replacement (i.e. the new population or RefSet will consist in the best *N* solutions selected from the previous RefSet members and the new *offspring* solutions), the enhanced scatter search uses a 1+1 replacement, similar to the strategy used in a very efficient evolutionary method, Differential Evolution [29]. This means that a RefSet member can only be replaced by a solution that has been generated combining by the former and another RefSet member. In other words, an *offspring* solution can only replace the RefSet member that generated it, and not any other. This strategy enhances diversity and prevents the search from premature stagnation by not allowing too similar solutions to be present in the RefSet at the same time. The “go-beyond” strategy to exploit combinations which explore promising directions has also been implemented. This strategy analyzes the search directions defined by a RefSet member and their *offspring*. If an *offspring* solution outperforms its corresponding RefSet member (i.e. the RefSet member that generates it), then the method considers that the explored direction is promising and a new solution is generated within such direction, exploring an area beyond the segment defined by the RefSet member and its *offspring* solution. The process is repeated until the new generated solutions do not outperform the previous ones and it favours intensification in the current iteration. Additionally, the use of memory is also exploited to select the most efficient initial points to perform local searches, to avoid premature convergence and to perturb solution vectors which are stuck in stationary points. More details about the enhanced scatter search scheme can be found in [30].

### Variable Neighbourhood Search (VNS)

Variable Neighbourhood Search is a trajectory-based metaheuristic for global optimization. It was introduced by Mladenović and Hansen [31] and has gained popularity in recent years in the field of global optimization. VNS performs a local search by evaluating the objective function around an incumbent solution and repeats the procedure visiting different neighbourhoods to locate different local optima, among which the global optimum is expected to be found. One of the key points of the algorithm is the strategy followed to change the current neighbourhood. VNS usually seeks a new neighbourhood by perturbing a set of decision variables using a distance criterion. Once a new solution has been created in the new neighbourhood, a new local search is performed. The typical scheme consists of visiting neighbourhoods close to the current one (i.e. perturbing a small set of solutions), until no further improvement is achieved. Then, more distant neighbourhoods are explored. Apart from this basic scheme, we have implemented advanced strategies to avoid cycles in the search (e.g. not repeating the perturbed decision variables in consecutive neighbourhood searches) in order to increase the efficiency when dealing with large-scale problems (e.g. by allowing a maximum number of perturbed decision variables, like in the Variable Neighbourhood Decomposition Search strategy [32]). We have also modified the search aggressiveness to locate high quality solutions (even if they are not the global optimum) in short computational times if required. Other heuristics, like the “go-beyond” strategy (explained above), that is used to exploit promising directions during the local search, have been adapted from other metaheuristics for continuous optimization [30].

### BayesFit

BayesFit is a Bayesian inference method for parameter estimation that uses Markov Chain Monte Carlo (MCMC) to sample the complete probability distributions of parameters. This accounts for both experimental error and model non-identifiability. It is available in the R version of MEIGO and has been adapted from the Python package BayesSB [33]. The sampling of the probability distributions uses a multi-start MCMC algorithm where the number of visits to a position in the parameter space is proportional to the posterior probability. The MCMC walk is punctuated by a Metropolis Hastings (M-H) criterion that allows more distant neighbourhoods to be explored, based on a probabilistic calculation.

### Cooperation

The cooperation scheme implemented in MEIGO is based on the following idea: to run, in parallel, several implementations or threads of an optimization algorithm, which may have different settings and/or random initializations, and exchange information between them. Since the nature of the optimization algorithms implemented in MEIGO is essentially different, we distinguish between eSS (the population based method) and VNS (the trajectory based method), following the classification proposed in [34] (currently there is no cooperation scheme for BayesFit): 

1. Information available for sharing: the best solution found and, optionally for eSS, the *RefSet*, which contains information about the diversity of solutions.

2. Threads that share information: all of them.

3. Frequency of information sharing: the threads exchange information at a fixed interval *τ*.

4. Number of concurrent programs: *η*.

Each of the *η* threads has a fixed degree of aggressiveness. “Conservative” threads have an emphasis on diversification (global search) and are used to increase the probability of finding a feasible solution, even if the parameter space is rugged or weakly structured. “Aggressive” threads have an emphasis on intensification (local search) and they speed up the calculations in smoother areas. Communication, which takes place at fixed time intervals, enables each thread to benefit from the knowledge gathered by the others. Thus this strategy has several degrees of freedom that have to be fixed: the time between communication (*τ*), the number of threads (*η*), and the strategy adopted by each thread. These adjustments should be chosen carefully depending on the particular problem we want to solve. Some guidelines for doing this can be found in [10] and in the Additional files 1, 2, 3, 4 and 5 accompanying this paper.

## Implementation

**Figure 1:**
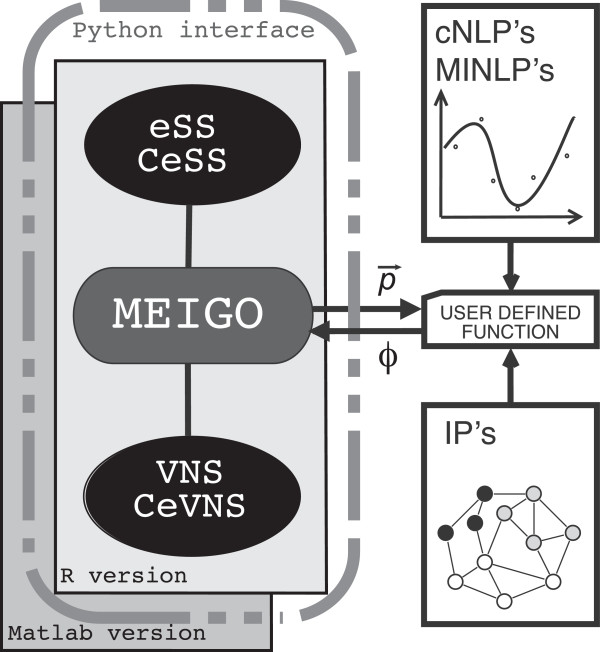
**MEIGO workflow.** Figure depicting the global structure of MEIGO.

MEIGO runs on Windows, Mac, and Linux, and provides implementations in both Matlab and R. So far, MEIGO includes: (i) eSS (Enhanced Scatter Search, [30]), for solving cNLP and MINLP problems, and (ii) VNS (Variable Neighbourhood Search), following the implementation described in [35], to solve IP problems (see Figure 1). The R version of MEIGO also includes the Bayesian parameter inference method BayesFit. Cooperative parallel versions (CeSS, CVNS), which can run on multicore PCs or clusters, are also included. Cooperation enhances the efficiency of the methods, not only in terms of speed, but also in terms of range: the threads running in parallel are completely independent so they can be customized to cover a wide range of search options, from aggressive to robust. In a sense the cooperation, as it has been designed, acts as a combination of different metaheuristics since each of the threads may present a different search profile. Four different kernel functions per method are included depending on the programming language chosen and the parallelization capabilities. Parallel computation in Matlab is carried out making use of the jpar tool [36]. Parallel computation in R can be performed using the package snowfall [37].

The methods implemented in MEIGO consider the objective functions to be optimized as black-boxes, with no requirements with respect to their structure. The user must provide a function that can be externally called for evaluation, accepting as input the variables to be estimated, and providing as output the objective value, *ϕ*, as a function of the input parameters. For constrained problems, the values of the constraints are also provided as output so that penalization functions can be calculated. For eSS and VNS, the user must define a set of compulsory fields (e.g. the name of the objective function, the bounds in the parameters, the maximum number of function evaluations). Further options take default values or can be changed. After each optimization, all the necessary results are stored in data files for further analysis with the tools provided by the host platforms. BayesFit is similarly robust to the form of the problem; in this case the likelihood function is provided by the user and this is incorporated into the calculation for the posterior probability for the parameter set, given the data.

Importantly, MEIGO is an open optimization platform in which other optimization methods can be implemented regardless of their nature (e.g. exact, heuristic, probabilistic, single-trajectory, population-based, etc.).

## Illustrative examples

To illustrate the capabilities of the methods presented here, a set of optimization problems, including cases from systems biology and bioinformatics, have been solved and are presented as case studies. The examples include (i) a set of state of the art benchmark cases for global optimization (from the Competition on Large Scale Global Optimization, 2012 IEEE World Congress on Computational Intelligence), (ii) a metabolic engineering problem based on a constraint-based model of *E. coli*, (iii) training of logic models of signaling networks to phospho-proteomic data [38], and (iv) an additional toy logic model [22] to compare BayesFit to eSS. The corresponding code for these examples is included in the distribution of the MEIGO software.

### Large-scale continuous global optimization benchmark

**Figure 2:**
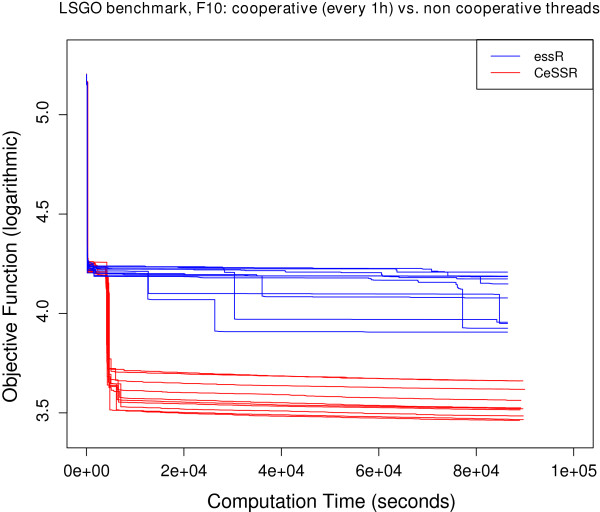
Convergence curves for f10 function.

**Figure 3:**
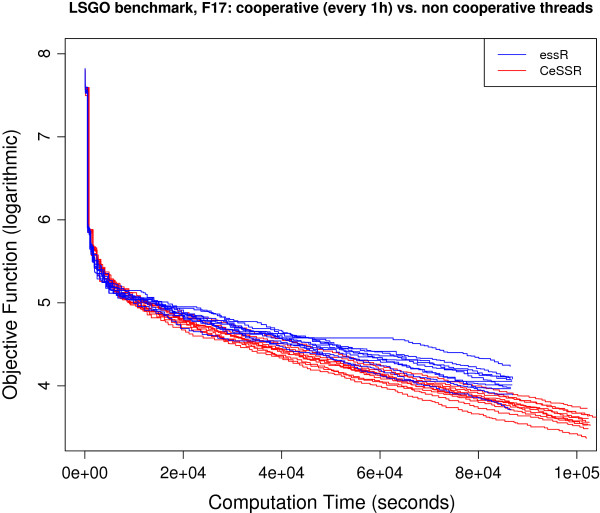
Convergence curves for f17 function.

**Figure 4:**
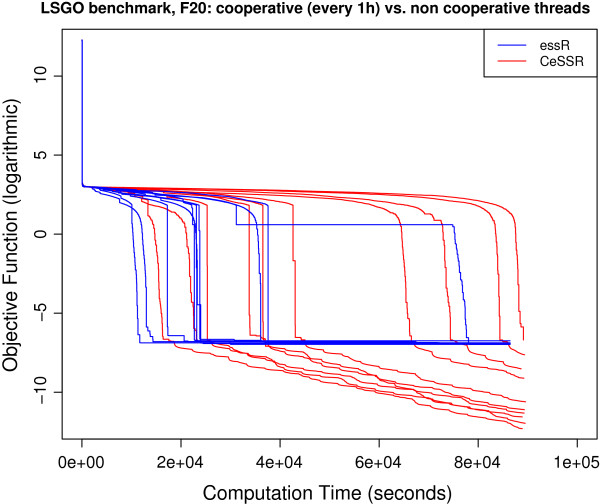
Convergence curves for f20 function.

These are benchmark functions used in the Special Session on Evolutionary Computation for Large Scale Global Optimization, which was part of the 2012 IEEE World Congress on Computational Intelligence (CEC@WCCI-2012). These objective functions can be regarded as state-of-the-art benchmark functions to test numerical methods for large-scale (continuous) optimization. Information about the functions as well as computer codes can be downloaded from http://staff.ustc.edu.cn/~ketang/cec2012/lsgo_competition.htm. Some of these functions were previously solved in [10] using CeSS, a cooperative version of the Enhanced Scatter Search metaheuristic implemented in Matlab and available within MEIGO. Large-scale calibration of systems biology models were also presented and solved in that paper. Here we present the solution of 3 of these functions (i.e. **f10**, **f17** and **f20**) using the R version of CeSS used by MEIGO. The convergence curves for the solution of these benchmark functions in R are coherent with those presented in [10], which were solved with Matlab, and the results are also competitive with the reference results for these functions presented in http://staff.ustc.edu.cn/~ketang/cec2012/lsgo_competition.htm. The convergence curves corresponding to these results are presented in Figures 2, 3 and 4.

### Integer optimization benchmark problems

A set of integer optimization problems arising in process engineering and coded in AMPL (*A Modeling Language for Mathematical Programming*) were solved using the Matlab version of VNS and making use of the AMPL-Matlab interface files provided by Dr. Sven Leyffer, available at http://www.mcs.anl.gov/~leyffer/macminlp/. VNS solved all the problems and, in some cases, achieved a better solution than the best reported one. A summary of the tested problems is presented in Table 1. These benchmarks have been solved using the Matlab version of MEIGO under Windows only, since the dynamic library to access AMPL files runs on Windows.

**Table 1:** Summary of solutions for integer programming problems

**Name**	**nvar**	**Ref.**	**Best reported**	**Best VNS**
			**solution**	**solution**
geartrain	4	[39]	7.78e-7	**2.70e-12**
mittelman	16	-	13.0	13.0
trimlon2	8	[40]	5.3	5.3
trimlon4	24		11.3	**8.3**
trimlon5	35		12.1	**10.6**

Data in boldface: solutions outperforming the best reported solution for that problem.

### Metabolic engineering example

In this section we illustrate the application of the VNS algorithm to a metabolic engineering problem. Here VNS was used to find a set of potential gene knock-outs that will maximize the production of a given metabolite of interest. The objective function is given by flux-balance analysis (FBA) where a steady-state model is simulated by means of linear programming (LP). The mathematical formulation is similar to that presented in [41]. FBA assumes that cells have a biological objective that is often considered as growth rate maximization, minimization of ATP consumption or both.

In this example we considered a small steady-state model from *E. coli* central carbon metabolism, available at http://gcrg.ucsd.edu/Downloads/EcoliCore. Here the metabolite of interest is succinate and we considered the biological objective as biomass maximization. To solve the inner FBA problem we used openCOBRA (http://opencobra.sourceforge.net/) with Gurobi as an LP solver (http://www.gurobi.com/). For the problem encoding, 5 integer variables were chosen as decision variables, one for each possible gene knock-out. Each of these variables was allowed to vary from 0 (no knock-out) to 52, the total number of possible genes to be knocked-out. Repeated KOs were filtered by the objective function.

**Figure 5:**
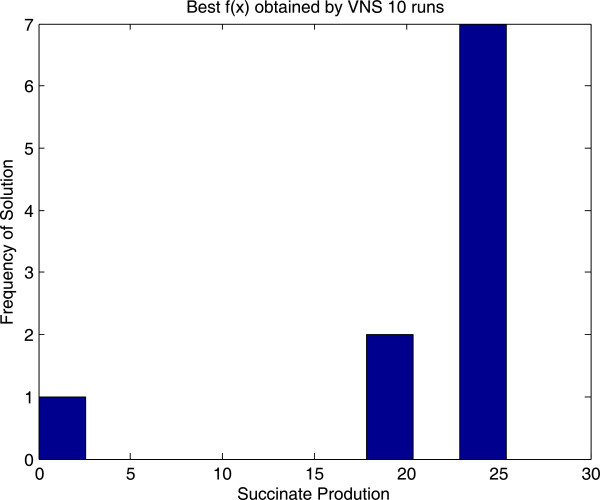
Histogram of the solutions obtained by VNS over 10 runs for the metabolic engineering example.

**Figure 6:**
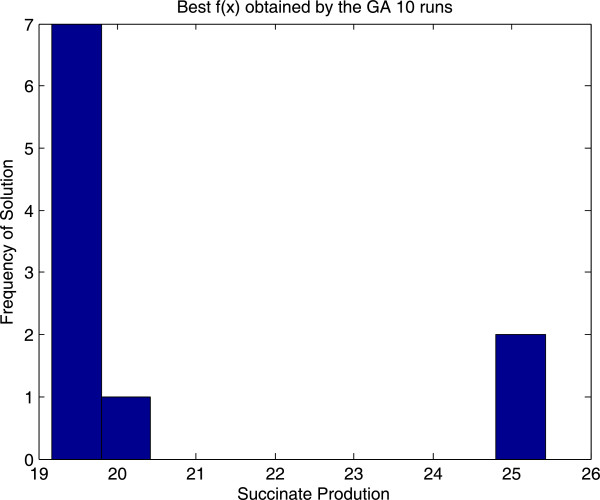
Histogram of the solutions obtained by the genetic algorithm over 10 runs for the metabolic engineering example.

Additionally we also implemented and solved the problem with a genetic algorithm from the Matlab Global Optimization Toolbox. The point here was to cross-check the VNS results, not to perform an extensive comparison between the performances of GA and VNS. However we found that for our particular problem and encoding, VNS achieved the optimal solution more often (see Figures 5 and 6). The Wilcoxon rank sum test with continuity correction for comparing means provides a p-value of 0.068 (or 0.021 if we remove the outlier VNS solution) showing that the solutions provided by VNS are significantly better. Please note that the GA was used out of the box (with default settings). Results can vary when using other encodings and further tuning of the search parameters. In any case, the purpose was to illustrate how this class of problems can be easily solved using VNS.

### Training of logic models of signalling networks to phospho-proteomic data

In this section we compare the performance of variable neighborhood search (VNS) and a discrete genetic algorithm (GA) implementation in training a logic model of a signalling network to phospho-proteomic data [38].

The problem is formulated as follows: one starts from a signed directed graph, containing the prior knowledge about a signaling network of interest. This graph contains directed edges among nodes (typically proteins) and their sign (activating or inhibitory). From this graph, one generates all possible AND and OR gates compatible with the graph. That means, if there are more than one edge arriving at a node, these are combined as OR and AND gates. Mathematically, this is encoded as an hyper graph, where edges with two or more inputs (hyperedges) represent a logical disjunction (AND gate). OR gates are encoded implicitly, by means of edges with only one input arriving at a node. See [38] for details.

To calibrate such models, the authors formulated the inference problem as a binary multi-objective problem, where the first objective corresponded to how well the model described the experimental data and the second consisted of a complexity penalty to avoid over-fitting:

(1) $$\theta (P)=\theta_{f}(P)+\alpha \cdot \theta_{s}(P)$$

where $\theta_{f}(P)=\frac{1}{n_{E}}\overset{s}{\underset{k=1}{\sum }}\overset{m}{\underset{l=1}{\sum }}\overset{n}{\underset{t=1}{\sum }}{\left(B_{k,l,t}^{M}(P)-B_{k,l,t}^{E}\right)}^{2}$ and $\theta_{s}(P)=\frac{1}{v_{e}^{s}}\overset{r}{\underset{e=1}{\sum }}v_{e}P_{e}$ such that *B*_*k*,*l*,*t*_(*P*)∈{0,1} is the value (0 or 1) as predicted by computation of the model’s logical steady state [42] and $B_{k,l,t}^{E}\in [\;0,1)$ is the data value for readout *l* at time *t* under the *k*th experimental condition. *θ*_*f*_(*P*) is the mean squared error and *α*·*θ*_*s*_(*P*) is the product between a tunable parameter *α* and a function denoting the model complexity (each hyper edge receives a penalty proportional to the number of inputs. E.g. an AND gates with 3 inputs is penalised 3 times as a single edge. OR gates arise implicitly from the combination of single input edges.).

Noticeably, the binary implementation of this problem contains redundant solutions in the search space. This can be addressed by compressing the search space into a reduced set containing only the smallest non-redundant combinations of hyperedges [38] (equivalent to the Sperner hypergraph). By doing this, the problem is transformed from a binary to an integer programming problem that was solved in [38] using a genetic algorithm.

Here, we implemented this benchmark by using the Matlab version of CellNetOptimizer (CNO or CellNOpt, available at http://www.cellnopt.org/downloads.html). The prior-knowledge network and data-set are also publicly available and thoroughly described at http://www.ebi.ac.uk/~cokelaer/cellnopt/data/ExtLiverPCB.html.

**Figure 7:**
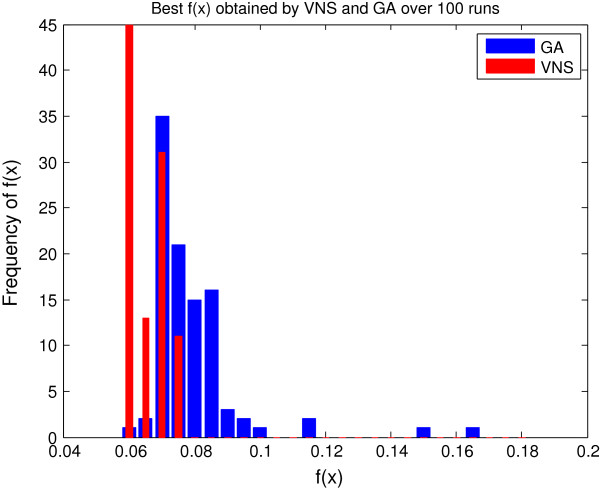
Histogram of solutions obtained by VNS and GA over 100 runs for the logic model example.

In order to assess the performance of both algorithms we solved each problem 100 times using VNS and the GA implementation from CNO. In the allowed time budget, VNS returned solutions that were on average better than those found by the GA (see Figure 7). The Welch Two Sample t-test for comparing means provides a p-value of 3.5·10^−14^, which clearly shows that VNS outperforms GA for this problem. Since both methods are sensitive to the tuning parameters, we tried to tune both algorithms fairly. Also, we note that the solution of this problem in its original, binary implementation can be solved using deterministic methods based either on Integer Linear Programming [43,44] or Answer Set Programming [45].

### Training of logic ODEs to in-silico generated data

**Figure 8:**
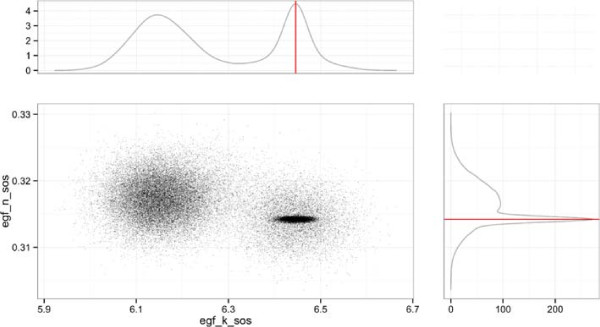
**The covariance in marginal posterior distributions between the parameters “egf_n_sos” and “egf_k_sos” computed by BayesFit.** The red lines in the marginal posterior distributions indicate the values of the parameters that produced the best fit to the data. The model and data are from [22].

Here, in order to demonstrate the additional information that can be derived from the probability distributions of optimized parameters, we compared the R implementations of BayesFit and eSS. The problem is again based on a logic model where, this time, the topology of the model is known and the goal is to optimize the parameters of the transfer functions used to generate a continuous simulation of the model. The parameters were optimized to reduce the distance between the model simulation and in silico generated data. This example is as described in section 6 from [22]; the only difference is that the model used here is the compressed model used to generate the in silico data in [22]. BayesFit produced a good fit to the data, comparable to that of eSS (Mean Squared Error: BayesFit, 0.007; eSS, 0.005). One of the advantages of estimating parameters by Bayesian inference is that parameter identifiability can be deduced from the marginal distributions for each parameter. For example Figure 8 shows two parameters of a single interaction between the species “egf” and “sos” in the model; these parameters *n* and *k*, control the shape of the transfer function between the two species [22]. From this figure, the covariation between the 2 parameters is evident. The best fit parameters (red line) lie in one region of high probability. However, there are additional correlated peaks in the marginal distributions of the two parameters, which suggests different parameter values could also produce a strong fit to the data.

## Conclusions

Here, we present MEIGO, a free, open-source and flexible package to perform global optimization in R, Matlab, and Python. It includes advanced metaheuristic methods. Furthermore, its modular nature (Figure 1), enables the connection to existing optimization methods.

## Availability and requirements

**Project name:** Metaheuristics for global optimization in systems biology and bioinformatics (MEIGO)**Project home page:**http://www.iim.csic.es/~gingproc/meigo.html**Operating system(s):** Windows, Linux, Mac OS X **Programming language:** Matlab 7.5 or higher and R 2.15 or higher **Licence:** GPLv3

## Competing interests

The authors declare that they have no competing interests.

## Authors’ contributions

JSR and JRB conceived, designed and coordinated the project. JAE implemented the metaheuristic methods included in MEIGO. AFV and DH developed and implemented the methods for parallel computation. JAE, AFV and DH performed the numerical computations for the metaheuristic methods. AMN and DPD implemented and performed the computational test of the bayesian method. TC designed and developed the Python wrapper and helped with many technical aspects. All authors contributed to the writing of the manuscript. All authors read and approved the final manuscript.

## Supplementary Material

Additional file 1 **MEIGO - Matlab’s users manual.** The file includes the user’s manual of the Matlab version of MEIGO. Click here for file

Additional file 2 **MEIGO - R’s user’s manual.** The file includes the users manual of the R version of MEIGO. Click here for file

Additional file 3 **Test of options for the integer programming benchmark problems.** The file includes the test carried out with the different search options to solve the integer programming benchmark problems as well as the extracted conclusions. Click here for file

Additional file 4 **MEIGO Matlab version source code and examples.** The file includes the source code of the Matlab version of MEIGO and the examples included in the users manual. Click here for file

Additional file 5 **MEIGO R version source code and examples.** The file includes the source code of the R version of MEIGO and the examples included in the users manual. Click here for file
