# Supplementary material for: MEIGO: an open-source software suite based on metaheuristics for global optimization in systems biology and bioinformatics
Source: BMC Bioinformatics. 2014 May 10;15:136. doi: 10.1186/1471-2105-15-136 (PMC4025564; doi:10.1186/1471-2105-15-136)
Supplement: Additional file 1 — MEIGO - Matlab’s users manual. The file includes the user’s manual of the Matlab version of MEIGO. [file 1471-2105-15-136-S1.pdf]

MEIGO: a software suite based on metaheuristics for global optimization in systems biology and bioinformatics
Matlab Version - User's guide

Jose A. Egea, David Henriques, Thomas Cokelaer,
Alejandro F. Villaverde, Julio R. Banga, Julio Saez-Rodriguez
saezrodriguez@ebi.ac.uk
julio@iim.csic.es
josea.egea@upct.es

October 21, 2013

Contents

1 Introduction **1**

2 Installing *MEIGO* **1**

2.1 Local solvers (mex files) compatibility list 1

3 Continuous and mixed-integer problems: Enhanced Scatter Search (*eSS*) **2**

3.1 Quick start: How to carry out an optimization with *eSS* 2

3.2 *eSS* usage 3

3.2.1 Problem definition 3

3.2.2 User options 4

3.2.3 Global options 5

3.2.4 Local options 5

3.2.5 Output 6

3.2.6 Guidelines for using *eSS* 7

3.2.7 Extra tool: *ess_multistart* 7

3.3 Application examples 8

3.3.1 Unconstrained problem 8

3.3.2 Constrained problem 9

3.3.3 Constrained problem with equality constraints 9

3.3.4 Mixed integer problem 11

3.3.5 Dynamic parameter estimation problem using *N2FB* 12

3.3.6 *ess_multistart* application 13

4 Integer optimization: Variable Neighbourhood Search (*VNS*) **13**

4.1 Quick start: How to carry out an optimization with *VNS* 15

4.2 *VNS* usage 15

4.2.1 Problem definition 15

4.2.2 *VNS* options 15

4.2.3 Output 16

4.2.4 Guidelines for using *VNS* 16

4.3 Application example 17

5 Parallel computation in *MEIGO* **17**

5.1 *jPar* installation 17

5.2 Use of *jPar* 18

5.3 Quick start: How to carry out an optimization with *CeSS* or *CeVNS* 18

5.4 Options and problem definition 19

5.4.1 Output 19
5.5 *CeSS* application example 20
5.6 *CeVNS* application example 21

Appendix A: List of options for eSS 23

Appendix B: List of options for VNS 24

References 25

1 Introduction

MEIGO is an optimization suite which implements metaheuristics for solving different nonlinear optimization problems in both continuous and integer domains arising in systems biology, bioinformatics and other areas. It consists of two main metaheuristics: the enhanced scatter search method, *eSS* (Egea et al., 2009, 2010) for continuous and mixed-integer problems, and the variable neighbourhood search metaheuristic (Mladenović and Hansen, 2010), for integer (combinatorial) optimization problems.

Both metaheuristics have been implemented in Matlab and can be invoked within the *MEIGO* framework. This manual describes the use of both methods (*eSS* and *VNS*), and their corresponding parallel versions based on a cooperative strategy (*CeSS* and *CVNS*).

2 Installing *MEIGO*

1. Download the MEIGO package from `http://www.iim.csic.es/~gingproc/meigo.html`.
2. Inside Matlab move to the package directory and add MEIGO to the Matlab path by running the script `install_MEIGO.m`.

Important: the Matlab implementations of eSS and CeSS make use of several local optimization solvers which were available in C or Fortran. We have developed mex files (dynamic link libraries) to call these solvers from Matlab. However, please note that they are only fully functional for the Windows operating system. Further, there are issues regarding the mex files compatibility for the 32 and 64 bits versions of Matlab. We provide a table below which illustrates which solvers run under which combination of Matlab and operating system.

2.1 Local solvers (mex files) compatibility list

The current distribution of MEIGO does not include mex files (binaries) for all the local solvers and every operating system (OS). Table 1 lists the currently working local solvers binaries for each OS. Note that if you have a 64-bit OS you can circumvent this by installing the 32-bit version of Matlab. Moreover for the case of IPOPT, instructions to obtain a compatible MEX file are available in `https://projects.coin-or.org/Ipopt/wiki/MatlabInterface`.

Important: the general conclusion from the compatibility table is that, if you want to have a fully functional version of eSS, you should use it with Matlab 32 bits version under Windows (tested with 32bits versions R2007-2011). This is especially important for parameter estimation problems, where the use of local solver n2fb results in very significant speed-ups.

3 Continuous and mixed-integer problems: Enhanced Scatter Search (*eSS*)

eSS is the Matlab implementation of the enhanced scatter search method (Egea et al., 2009, 2010) which is part of the *MEIGO* toolbox for global optimization in bioinformatics. It is a metaheuristic which seeks the global minimum of mixed-integer nonlinear programming (MINLP) problems specified by

*Tested under Matlab 32 bits versions R2007b-R2011. Later versions might present issues related with lack of backwards compatibility of the MEX files.

†Requires Matlab Optimization Toolbox.

	Windows		Linux		MAC OS X
Local solver	Matlab 32-bits	Matlab 64-bits	Matlab 32-bits	Matlab 64-bits	Matlab
N2FB*	yes	no	yes	no	no
IPOPT*	yes	no	no	no	no
FMINSEARCH †	yes	yes	yes	yes	yes
DHC	yes	yes	yes	yes	yes
HOOKE	yes	yes	yes	yes	yes
NOMAD	yes	yes	yes	yes	yes
SOLNP	yes	yes	yes	yes	yes
LSQNONLIN †	yes	yes	yes	yes	yes
FMINCON †	yes	yes	yes	yes	yes

Table 1: Compatibility table of local optimization solvers. Solvers implemented as MEX files might not run in all the operating systems.

$$\min_x f(x, p_1, p_2, ..., p_n)$$

subject to

$$\begin{aligned} c_{eq} &= 0 \\ c_L \le c(x) &\le c_U \\ x_L \le x &\le x_U \end{aligned}$$

where x is the vector of decision variables, and x_L and x_U its respective bounds. $p_1, \ldots, p_n$ are optional extra input parameters to be passed to the objective function (see examples in sections 3.3.3, 3.3.5). c_{eq} is a set of equality constraints. $c(x)$ is a set of inequality constraints with lower and upper bounds, c_L and c_U. Finally, $f(x, p_1, p_2, ..., p_n)$ is the objective function to be minimized.

3.1 Quick start: How to carry out an optimization with *eSS*

- Start Matlab
- Add **MEIGO** Toolbox to the Matlab path. *.
- Define your problem and options similarly to this scheme (see the following sections)

```
problem.f='ex1';                          %mfile containing the objective function
problem.x_L=-1*ones(1,2);                 %lower bounds
problem.x_U=ones(1,2);                    %upper bounds
opts.maxeval=500;
opts.local.solver='dhc';
```

- Type: `Results=MEIGO(problem,opts, 'ESS')`. If your problem has additional constant parameters to be passed to the objective function, they are declared as input parameters after “opts” (e.g., type `Results=MEIGO(problem,opts,'ESS',p1,p2)` if your model has two extra input parameters, p_1 and p_2).

*The *MEIGO* package can be downloaded for installation at `http://www.iim.csic.es/~gingproc/meigo.html`

Regarding the objective function, the input parameter is the decision vector (with extra parameters $p_1, p_2, \ldots, p_n$ if they were defined before calling the solver). The objective function must provide a scalar output parameter (the objective function value) and, for constrained problems, a second output parameter, which is a vector containing the values of the constraints. For problems containing equality constraints (= 0), they must be defined before the inequality constraints. Some examples are provided in section 3.3. For a quick reference, consider the following example which will be later extended in section 3.3.3.

$$\min_x f(x) = -x_4$$

subject to

$$\begin{aligned} x_4 - x_3 + x_2 - x_1 + k_4 x_4 x_6 &= 0 \\ x_1 - 1 + k_1 x_1 x_5 &= 0 \\ x_2 - x_1 + k_2 x_2 x_6 &= 0 \\ x_3 + x_1 - 1 + k_3 x_3 x_5 &= 0 \\ x_5^{0.5} + x_6^{0.5} &\leq 4 \\ 0 \leq x_1, x_2, x_3, x_4 &\leq 1 \\ 0 \leq x_5, x_6 &\leq 16 \end{aligned}$$

with k_1, k_2, k_3, k_4 being extra parameters defined before calling the solver. The objective function for this problem would be:

example of objective function

```
function [f,g]=ex3(x,k1,k2,k3,k4)
f=-x(4);

%Equality constraints. Declared BEFORE inequality constraints
g(1)=x(4)-x(3)+x(2)-x(1)+k4*x(4).*x(6);
g(2)=x(1)-1+k1*x(1).*x(5);
g(3)=x(2)-x(1)+k2*x(2).*x(6);
g(4)=x(3)+x(1)-1+k3*x(3).*x(5);

%Inequality constraint
g(5)=x(5).^0.5+x(6).^0.5;

return
```

This objective function can be invoked like this (let us assume that x has dimension 6 and we define the 4 extra input parameters k_1 to k_4)

```
[f]=ex3(ones(1,6),0.2,0.5,-0.1,0.9)
```

3.2 *eSS* usage

3.2.1 Problem definition

In order to solve an optimization problem with *eSS*, a structure (named **problem** here) containing the following fields must be defined:

- **f**: String containing the name of the objective function.
- **x_L**: Vector containing the lower bounds of the variables.

- **x_U**: Vector containing the upper bounds of the variables.

Besides, there are two optional fields

- **x_0**: Vector or matrix containing the given initial point(s).
- **f_0**: Function values of the initial point(s). These values MUST correspond to feasible points.
- **vtr**: Objective function value to be reached.

If the problem contains additional constraints and/or integer or binary variables, the following fields should also be defined:

- **neq***: Number of equality (= 0) constraints.
- **c_L**: Vector defining the lower bounds of the inequality constraints.
- **c_U**: Vector defining the upper bounds of the inequality constraints.
- **int_var**†: Number of integer variables.
- **bin_var**†: Number of binary variables.

3.2.2 User options

The user may define a set of different options related to the optimization problem. They are defined in another structure (named **opts** here) which has the following fields:

- **maxeval**: Maximum number of function evaluations (default: 1000).
- **maxtime**: Maximum CPU time in seconds (default: 60).
- **iterprint**: Print information on the screen after each iteration. 0: Deactivated; 1: Activated (default: 1).
- **plot**: Plots convergence curves. 0: Deactivated; 1: Real Time; 2: Final results (default: 0).
- **weight**: Weight that multiplies the penalty term added to the objective function in constrained problems (default: 10^6).
- **log_var**: Indexes of the variables which will be analyzed using a logarithmic distribution instead of an uniform one‡ (default: empty). See an example in Section 3.3.5.
- **tolc**: Maximum constraint violation allowed. This is also used as a tolerance for the local search (default: 10^{-5}).
- **prob_bound**: Probability (0-1) of biasing the search towards the bounds. 0: Never bias to bounds; 1: Always bias to bounds (default: 0.5).
- **inter_save**: Saves results of intermediate iterations in **eSS_report.mat**. Useful for very long runs. 0: deactivated; 1: activated (default: 0).

*In problems with equality constraints they must be declared first before inequality constraints (See example 3.3.3)

†For mixed integer problems, the variables must be defined in the following order: [cont., int., bin.].

‡Useful when the bounds of a decision variables have different orders of magnitude and they are both positive.

3.2.3 Global options

A set of options related to the global search phase of the algorithm may also be defined within the structure **opts**:

- **dim_refset**: Number of elements d in *RefSet* (default: *"auto"*, $\frac{d^2-d}{10 \cdot nvar} \geq 0$).
- **ndiverse**: Number of solutions generated by the diversificator in the initial stage (default: *"auto"*, $10 \cdot nvar$).
- **combination**: Type of combination of *RefSet* elements. 1: Hyper-rectangles combinations; 2: Linear combinations (default: 1).

3.2.4 Local options

eSS is a global optimization method which performs local searches from selected initial points to accelerate the convergence to optimal solutions. Some options regarding the local search can be defined in a sub-structure within the options structure (named **opts.local** here), which has the following fields:

- **solver**: Local solver to perform the local search. Solvers available (names must be introduced as strings):
  - *fmincon*:‡ Sequential quadratic programming method (The MathWorks™, 2008).
  - *solnp*: the SQP method¶.
  - *fminsearch*:‡. Modification of the Simplex-based method implemented in Matlab (The MathWorks™, 2008) to handle bound constraints, by John D'Errico.
  - *dhc*: Direct search method (de la Maza and Yuret, 1994).
  - *hooke*: Hooke & Jeeves direct search method (Hooke and Jeeves, 1961)‖.
  - *n2fb*: Specific method for non-linear least squares problems (Dennis et al., 1981).
  - *lsqnonlin*:‡ Another method for non-linear least squares problems (The MathWorks™, 2008).
  - *ipopt*: Interior point method (Wächter and Biegler, 2006).
- **tol**: Level of tolerance in local search. 1: Relaxed; 2: Medium; 3: Tight (default: 2 in intermediate searches and 3 in the final stage).
- **iterprint**: Print each iteration of local solver on screen (only for local solvers that allow it). 0: Deactivated; 1: Activated (default: 0).
- **n1**: Number of iterations before applying the local search for the first time (default: 1).
- **n2**: Number of function iterations in the global phase between two consecutive local searches (default: 10).
- **finish**: Applies local search to the best solution found once the optimization is finished (default: same as *opts.local.solver*).
- **bestx**: If activated (i.e., positive value), applies the local search only when the algorithm finds a solution better than the current best solution. 0: Deactivated; 1: Activated (default: 0).

‡Requires the Matlab Optimization Toolbox for its use.

¶The original Matlab code can be found at `http://www.stanford.edu/~yyye/matlab/` by Ye (1987)

‖C.T. Kelley's Matlab code is used (`http://www4.ncsu.edu/~ctk/darts/hooke.m`)

- **balance**: Balances between quality (=0) and diversity (=1) for choosing initial points for the local search (default 0.5).

Note that, for some problems, the local search may be inefficient, spending a high computation time to provide low quality solutions. This is the case of many noisy or ill-posed problems. In these instances, the local search may be deactivated by user by defining the value of the field **solver** as zero.

When using *n2fb* (or *dn2fb*) and *lsqnonlin* as local solvers, the objective function value must be formulated as the square of the sum of differences between the experimental and predicted data (i.e., $\sum_{i=1}^{ndata}(yexp_i - yteor_i)^2$). Besides, a third output argument must be defined in the objective function: a vector containing those residuals (i.e., $R = [(yexp_1 - yteor_1), (yexp_2 - yteor_2), \ldots, (yexp_{ndata} - yteor_{ndata})]$). In Section 3.3.5 an application example illustrates the use of these local methods.

3.2.5 Output

eSS's output is a structure (called **Results** here) containing the following fields:

- **Results.fbest**: Best objective function value found after the optimization.
- **Results.xbest**: Vector providing the best function value found.
- **Results.cpu_time**: CPU Time (in seconds) consumed in the optimization.
- **Results.f**: Vector containing the best objective function value after each iteration.
- **Results.x**: Matrix containing the best vector after each iteration.
- **Results.time**: Vector containing the CPU time consumed after each iteration.
- **Results.neval**: Vector containing the number of evaluations after each iteration.
- **Results.numeval**: Total number of function evaluations.
- **Results.local_solutions**: Matrix of local solutions found.
- **Results.local_solutions_values**: Function values of the local solutions.
- **Results.Refset.x**: Matrix of solutions in the final *Refset* after the optimization.
- **Results.Refset.f**: Objective function values of the final *Refset* members after the optimization.
- **Results.Refset.fpen**: Penalized objective function values of the final *Refset* members after the optimization. The values for feasible solutions will coincide with their corresponding **Results.Refset.f** values.
- **Results.Refset.const**: Matrix containing the values of the constraints (those whose bounds are defined in **problem.c_L** and/or **problem.c_U**) for each of the solutions in the final *Refset* after the optimization.
- **Results.end_crit**: Criterion to finish the optimization:
  - 1: Maximum number of function evaluations achieved.
  - 2: Maximum allowed CPU time achieved.
  - 3: Value to reach achieved.

The structures **Results**, **problem** and **opts** are saved in a file called `ess_report.mat`.

3.2.6 Guidelines for using *eSS*

Although *eSS* default options have been chosen to be robust for a high number of problems, the tuning of some parameters may help increase the efficiency for a particular problem. Here is presented a list of suggestions for parameter choice depending on the type of problem the user has to face.

- If the problem is likely to be convex, an early local search can find the optimum in short time. For that it is recommended to set the parameter **opts.local.n1 = 1**. Besides, setting **opts.local.n2 = 1** too, the algorithm increases the local search frequency, becoming an "intelligent" multistart.
- When the bounds differ in several orders of magnitude, the decision variables indexes may be included in **log_var**.
- For problems with discontinuities and/or noise, the local search should either be deactivated or performed by a direct search method. In those cases, activating the option **opts.local.bestx = 1** may help reduce the computation time wasted in useless local searches by performing a local search only when the best solution found has been improved.
- When the function values are very high in absolute value, the weight (**opts.weight**) should be increased to be at least 3 orders of magnitude higher than the mean function value of the solutions found.
- When the search space is very large compared to the area in which the global solution may be located, a first investment in diversification may be useful. For that, a high value of **opts.ndiverse** can help finding good initial solutions to create the initial *RefSet*. A preliminary run with aggressive options can locate a set of good initial solutions for a subsequent optimization with more robust settings. This aggressive search can be performed by reducing the size of the *RefSet* (**opts.dim_refset**). A more robust search is produced increasing the *RefSet* size.
- If local searches are very time-consuming, their tolerance can be relaxed by reducing the value of **opts.local.tol** not to spend a long time in local solution refinements.
- When there are many local solutions close to the global one, the option **opts.local.balance** should be set close to 0.

3.2.7 Extra tool: *ess_multistart*

This tool allows the user to perform a multistart optimization procedure with any of the local solvers implemented in *eSS* using the same problem declaration. *ess_multistart* can be invoked within *MEIGO* using the same structure setting the third input parameter as `'MULTISTART'`.

```
>> Results_multistart=MEIGO(problem,opts,'MULTISTART',p1,p2,...)
```

The structure **problem** has the same fields as in *eSS* (except **problem.vtr** which does not apply here).

The structure **opts** has only a few fields compared with *eSS* (i.e.,**opts.ndiverse**, **opts.local.solver**, **opts.iterprint**, **opts.local.tol** and **opts.local.iterprint**). They all work like in *eSS* except **opts.ndiverse**, which indicates the number of initial points chosen for the multistart procedure. A histogram with the final solutions obtained and their frequency is presented at the end of the procedure.

The output structure **Results_multistart** contains the following fields:

- **.fbest**: Best objective function value found after the multistart optimization.

- **.xbest**: Vector providing the best function value.
- **.x0**: Matrix containing the vectors used for the multistart optimization.
- **.f0**: Vector containing the objective function values of the vectors in **Results_multistart.x0**.
- **.func**: Vector containing the objective function values obtained after every local search.
- **.xxx**: Matrix containing the vectors provided by the local optimizations.
- **.no_conv**: Matrix containing the initial points that did not converge to any solution.
- **.nfuneval**: Matrix containing the number of function evaluations performed in every optimization.

The structures **problem**, **opts** and **Results_multistart** are saved in a file called `Results_multistart.mat`.

3.3 Application examples

In this section we will illustrate the usage of *eSS* within *MEIGO* for solving different instances.

3.3.1 Unconstrained problem

$$\min_x f(x) = 4x_1^2 - 2.1x_1^4 + 1/3x_1^6 + x_1x_2 - 4x_2^2 + 4x_2^4$$

subject to

$$-1 \leq x_1, x_2 \leq 1$$

The objective function is defined in `ex1.m`. Note that being an unconstrained problem, there is only one output argument, f.

ex1.m script

```
function F=ex1(x)
F=4*x(1).*x(1)-2.1*x(1).^4+1/3*x(1).^6+x(1).*x(2)-4*x(2).*x(2)+4*x(2).^4;
return
```

The solver is called in `main_ex1.m`. This problem has two known global optima in $x^* = (0.0898, -0.7127)$ and $x^* = (-0.0898, 0.7127)$ with $f(x^*) = -1.03163$.

Options set:

- Maximum number of function evaluations set to 500.
- Maximum number of initial diverse solutions set to 40.
- Local solver chosen: *dhc*.
- Local solver for final refinement: *fmincon*.
- Show the information provided by local solvers on screen.

main_ex1.m script

```
%========================= PROBLEM SPECIFICATIONS ===========================
problem.f='ex1';                                %mfile containing the objective function
problem.x_L=-1*ones(1,2);                       %lower bounds
problem.x_U=ones(1,2);                          %upper bounds

opts.maxeval=500;
opts.ndiverse=40;
opts.local.solver='dhc';
opts.local.finish='fmincon';
opts.local.iterprint=1;
%========================= END OF PROBLEM SPECIFICATIONS =====================

Results=MEIGO(problem,opts,'ESS');
```

3.3.2 Constrained problem

$$\min_x f(x) = -x_1 - x_2$$

subject to

$$\begin{aligned} x_2 &\leq 2x_1^4 - 8x_1^3 + 8x_1^2 + 2 \\ x2 &\leq 4x_1^4 - 32x_1^3 + 88x_1^2 - 96x_1 + 36 \\ 0 &\leq x_1 \leq 3 \\ 0 &\leq x_2 \leq 4 \end{aligned}$$

The objective function is defined in `ex2.m`. Note that being a constrained problem, there are two output arguments, f and g.

ex2.m script

```
function [F,g]=ex2(x)
F=-x(1)-x(2);
g(1)=x(2)-2*x(1).^4+8*x(1).^3-8*x(1).^2;
g(2)=x(2)-4*x(1).^4+32*x(1).^3-88*x(1).^2+96*x(1);
return
```

The solver is called in `main_ex2.m`. The global optimum for this problem is located in $x^* = [2.32952, 3.17849]$ with $f(x^*) = -5.50801$.

Options set:

- Maximum number of function evaluations set to 750.
- Increase frequency of local solver calls. The first time the solver is called after 2 iterations. From that moment, the local solver will be called every 3 iterations.

3.3.3 Constrained problem with equality constraints

$$\min_x f(x) = -x_4$$

main_ex2.m script

```
%========================= PROBLEM SPECIFICATIONS ===========================
problem.f='ex2';                         %mfile containing the objective function
problem.x_L=[0 0];                       %lower bounds
problem.x_U=[3 4];                       %upper bounds
problem.c_L=[-inf -inf];
problem.c_U=[2 36];

opts.maxeval=750;
opts.local.n1=2;
opts.local.n2=3;
%========================= END OF PROBLEM SPECIFICATIONS =====================
Results=MEIGO(problem,opts,'ESS');
```

subject to

$$
\begin{aligned}
x_4 - x_3 + x_2 - x_1 + k_4 x_4 x_6 &= 0 \\
x_1 - 1 + k_1 x_1 x_5 &= 0 \\
x_2 - x_1 + k_2 x_2 x_6 &= 0 \\
x_3 + x_1 - 1 + k_3 x_3 x_5 &= 0 \\
x_5^{0.5} + x_6^{0.5} &\leq 4 \\
0 \leq x_1, x_2, x_3, x_4 &\leq 1 \\
0 \leq x_5, x_6 &\leq 16
\end{aligned}
$$

with $k_1 = 0.09755988$, $k_3 = 0.0391908$, $k_2 = 0.99 \cdot k_1$ and $k_4 = 0.9 \cdot k_3$. The objective function is defined in `ex3.m`. Note that equality constraints must be declared before inequality constraints. Parameters $k_1, \ldots, k_4$ are passed to the objective function through the main script, therefore they do not have to be calculated in every function evaluation. See the input arguments below.

ex3.m script

```
function [f,g]=ex3(x,k1,k2,k3,k4)
f=-x(4);

%Equality constraints
g(1)=x(4)-x(3)+x(2)-x(1)+k4*x(4).*x(6);
g(2)=x(1)-1+k1*x(1).*x(5);
g(3)=x(2)-x(1)+k2*x(2).*x(6);
g(4)=x(3)+x(1)-1+k3*x(3).*x(5);

%Inequality constraint
g(5)=x(5).^0.5+x(6).^0.5;

return
```

The solver is called in `main_ex3.m`. The global optimum for this problem is located in $x^* = [0.77152, 0.516994, 0.204189, 0.388811, 3.0355, 5.0973]$ with $f(x^*) = -0.388811$.

Options set:

- Number of equality constraints set to 4 in `problem.neq`.
- Fields `problem.c_L` and `problem.c_U` only contain bounds for inequality constraints.
- Maximum computation time set to 5 seconds.
- Local solver chosen: *solnp*.
- Parameters $k_1, \ldots, k_4$ are passed to the main routine as input arguments.

main_ex3.m script

```
%========================= PROBLEM SPECIFICATIONS ===========================
problem.f='ex3';
problem.x_L=[0 0 0 0 0 0];
problem.x_U=[1 1 1 1 16 16];
problem.neq=4;
problem.c_L=-inf;
problem.c_U=4;

opts.maxtime=5;
opts.local.solver='solnp';
%========================= END OF PROBLEM SPECIFICATIONS =====================
k1=0.09755988;
k3=0.0391908;
k2=0.99*k1;
k4=0.9*k3;

[Results]=MEIGO(problem,opts,'ESS',k1,k2,k3,k4);
```

3.3.4 Mixed integer problem

$$\min_x f(x) = x_2^2 + x_3^2 + 2x_1^2 + x_4^2 - 5x_2 - 5x_3 - 21x_1 + 7x_4$$

subject to

$$\begin{aligned} x_2^2 + x_3^2 + x_1^2 + x_4^2 + x_2 - x_3 + x_1 - x_4 &\leq 8 \\ x_2^2 + 2x_3^2 + x_1^2 + 2x_4^2 - x_2 - x_4 &\leq 10 \\ 2x_2^2 + x_3^2 + x_1^2 + 2x_2 - x_3 - x_4 &\leq 5 \\ 0 \leq x_i \leq 10 \quad \forall i &\in [1, \ldots, 4] \end{aligned}$$

Integer variables: x_2, x_3 and x_4. In the function declaration (`ex4.m`) they must have the last indexes.

ex4.m script

```
function [F,g]=ex4(x)
F = x(2)^2 + x(3)^2 + 2.0*x(1)^2 + x(4)^2 - 5.0*x(2) - 5.0*x(3) - 21.0*x(1) + 7.0*x(4);
g(1) = x(2)^2 + x(3)^2 + x(1)^2 + x(4)^2 + x(2) - x(3) + x(1) - x(4);
g(2) = x(2)^2 + 2.0*x(3)^2 + x(1)^2 + 2.0*x(4)^2 - x(2) - x(4);
g(3) = 2.0*x(2)^2 + x(3)^2 + x(1)^2 + 2.0*x(2) - x(3) - x(4);
return
```

The solver is called in `main_ex4.m`. The global optimum for this problem is located in $x^* = [2.23607, 0, 1, 0]$ with $f(x^*) = -40.9575$.

Options set:

- An initial point is specified.
- The number of integer variables is specified (mandatory).
- No local solver for mixed-integer problems is available in *MEIGO*.
- Stop criterion determined by the CPU time (2 seconds).

main_ex4.m script

```
%========================= PROBLEM SPECIFICATIONS ===========================
problem.f='ex4';
problem.x_L=[0 0 0 0];
problem.x_U=[10 10 10 10];
problem.x_0=[3 4 5 1];
problem.int_var=3;
problem.c_L=[-inf -inf -inf ];
problem.c_U=[8 10 5];

opts.local.solver=0;
opts.maxtime=2;
%========================= END OF PROBLEM SPECIFICATIONS =====================

Results=MEIGO(problem,opts,'ESS');
```

3.3.5 Dynamic parameter estimation problem using *N2FB*

Here we will illustrate the use of *eSS* within *MEIGO* using *N2FB* as local solver. In particular, the problem considered is the isomerization of α-pinene (Rodríguez-Fernández et al., 2006).

$$\min_{p} J = \sum_{j=1}^{5}\sum_{i=1}^{8}(y_j(p,t_i) - \tilde{y}_{ji})^2$$

subject to the system dynamics

$$\begin{aligned}
\frac{dy_1}{dt} &= -(p_1+p_2)y_1 \\
\frac{dy_2}{dt} &= p_1 y_1 \\
\frac{dy_3}{dt} &= p_2 y_1 - (p_3+p_4)y_3 + p_5 y_5 \\
\frac{dy_4}{dt} &= p_3 y_3 \\
\frac{dy_5}{dt} &= p_4 y_3 - p_5 y_5
\end{aligned}$$

and subject to parameter bounds

$$0 \le x_i \le 1 \quad \forall i \in [1,\ldots,5]$$

In order to use *N2FB* as local solver, in the script `ex5.m` there must be three output arguments: apart from the objective function and the constraints (empty in this case), a vector R containing the residuals must be defined. The Matlab routine *ode15s* for stiff ODE systems is used to perform the numerical integration.

The solver is called in `main_ex5.m`. The global optimum for this problem is located in $\mathbf{p}^* = [5.93\cdot10^{-5}, 2.96\cdot10^{-5}, 2.0\cdot10^{-5}, 2.75\cdot10^{-4}, 4.00\cdot10^{-5}]$, with $f(\mathbf{p}^*) = 19.88$.

Options set:

- An initial point is specified.
- Maximum number of evaluations is 1000.
- All the variables are declared as *log_var*.

ex5.m script

```
function [J,g,R]=ex5(x,texp,yexp)

[tout,yout] = ode15s(@ex5_dynamics,texp,[100 0 0 0 0],[],x);

R=(yout-yexp);
R=reshape(R,numel(R),1);


J = sum(sum((yout-yexp).^2));
g=0;
return

%***********************************************
%Function of the dynamic system
function dy=ex5_dynamics(t,y,p)

dy=zeros(5,1);  %Initialize the state variables


dy(1)=-(p(1)+p(2))*y(1);
dy(2)=p(1)*y(1);
dy(3)=p(2)*y(1)-(p(3)+p(4))*y(3)+p(5)*y(5);
dy(4)=p(3)*y(3);
dy(5)=p(4)*y(3)-p(5)*y(5);

return
%***********************************************
```

- We choose the *N2FB* local solver.
- Save results in a file after every iteration.

3.3.6 *ess_multistart* application

An application of *ess_multistart* within *MEIGO* on the problem *ex3* using *solnp* as local solver is presented in the script `main_multistart_ex3.m`. The number of initial points chosen is 25.

4 Integer optimization: Variable Neighbourhood Search (*VNS*)

VNSm is a Matlab implementation of the Variable Neighbourhood Search (VNS) metaheuristic which is part of the *MEIGO* toolbox for global optimization in bioinformatics. VNS was first proposed by Mladenović and Hansen (1997) for solving combinatorial and/or global optimization problems. The method guides a trial solution to search for an optimum in a certain area. After this optimum is located, the trial solution is perturbed to start searching in a new area (or neighbourhood). New neighbourhoods are defined following a distance criterion in order to achieve a good diversity in the search. Different variants of the method have been published in recent years in order to adapt it to different types of problems (Mladenović and Hansen, 2010). VNS implements some of this variants by means of different tunning parameters.

VNS seeks the global minimum of integer programming (IP) problems specified by

$$\min_x f(x, p_1, p_2, ..., p_n)$$

subject to

main_ex5.m script

```
%======================== PROBLEM SPECIFICATIONS ===========================
problem.f='ex5';

problem.x_L=zeros(1,5);
problem.x_U=ones(1,5);

problem.x_0=0.5*ones(1,5);
opts.maxeval=1e3;
opts.log_var=[1:5];
opts.local.solver='n2fb';
opts.inter_save=1;
%======================== END OF PROBLEM SPECIFICATIONS =====================
%time intervals

texp=[0 1230 3060 4920 7800 10680 15030 22620 36420];

% Distribution of species concentration
 %      y(1)    y(2)    y(3)    y(4)    y(5)

 yexp=[ 100.0  0.0  0.0  0.0     0.0
     88.35    7.3     2.3     0.4     1.75
        76.4    15.6     4.5     0.7     2.8
        65.1    23.1     5.3     1.1     5.8
        50.4    32.9     6.0     1.5     9.3
        37.5    42.7     6.0     1.9    12.0
        25.9    49.1     5.9     2.2    17.0
        14.0    57.4     5.1     2.6    21.0
         4.5    63.1     3.8     2.9    25.7 ];


Results=MEIGO(problem,opts,'ESS',texp,yexp);
```

main_multistart_ex3.m script

```
%======================== PROBLEM SPECIFICATIONS ===========================
problem.f='ex3';
problem.x_L=[0 0 0 0 0 0];
problem.x_U=[1 1 1 1 16 16];
problem.neq=4;
problem.c_L=-inf;
problem.c_U=4;

opts.ndiverse=25;

opts.local.solver='solnp';
opts.local.iterprint=1;
opts.local.tol=2;
%=====================================================================
k1=0.09755988;
k3=0.0391908;
k2=0.99*k1;
k4=0.9*k3;

Results_multistart=MEIGO(problem,opts,'MULTISTART',k1,k2,k3,k4);
```

$$x_L \leq x \leq x_U$$

where x is the vector of (integer) decision variables, and x_L and x_U its respective bounds. $p_1, \ldots, p_n$ are optional extra input parameters to be passed to the objective function, f, to be minimized.

The current *VNS* version does not handle constraints apart from bound constrained, so that the user should formulate his/her own method (i.e., a penalty function) to solve constrained problems.

4.1 Quick start: How to carry out an optimization with *VNS*

- Start Matlab
- Run the script install_MEIGO.m, in order to add **MEIGO** * to the Matlab path.
- Define your problem and options (see the following sections)
- Type: `Results=MEIGO(problem,opts, 'VNS')`. If your problem has additional constant parameters to be passed to the objective function, they are declared as input parameters after "opts" (e.g., type `Results=MEIGO(problem,opts,'VNS',p1,p2)` if your model has two extra input parameters, p_1 and p_2).

Regarding the objective function, the input parameter is the decision vector (with extra parameters $p_1, p_2, \ldots, p_n$ if they were defined before calling the solver). The objective function must provide a scalar output parameter (the objective function value).

4.2 *VNS* usage

4.2.1 Problem definition

A structure named **problem** containing the following fields must be defined for running *VNS*:

- **f**: String containing the name of the objective function.
- **x_L**: Vector containing the lower bounds of the variables.
- **x_U**: Vector containing the upper bounds of the variables.
- **x_0**: Vector containing the initial solution to start the search. If this field is not defined then the algorithm will choose an initial point randomly chosen within the bounds.

4.2.2 *VNS* options

The user may define a set of different options related to the integer optimization problem. They are defined in another structure (named **opts** here) which has the following fields.

- **maxeval**: Maximum number of function evaluations (default: 1000).
- **maxtime**: Maximum CPU time in seconds (default: 60).
- **maxdist**: Percentage of the problem dimension which will be perturbed in the furthest neighborhood (vary between 0-1, default: 0.5).
- **use_local**: Uses local search (1) or not (0). Default:1.

The following options only apply when the local search is activated (i.e., opts.use_local=1)

- **aggr**: Aggressive search. The local search is only applied when the best solution has been improved (1=aggressive search, 0=non-aggressive search, default:0).

*The *MEIGO* package can be downloaded for installation at `http://www.iim.csic.es/~gingproc/meigo.html`

- **local_search_type**: Applies a first (=1) or a best (=2) improvement scheme for the local search (Default: 1).
- **decomp**: Decompose the local search (=1) using only the variables perturbed in the global phase. Default: 1.

4.2.3 Output

VNS output is a structure (called **Results** here) containing the following fields:

- **Results.fbest**: Best objective function value found after the optimization.
- **Results.xbest**: Vector providing the best function value found.
- **Results.cpu_time**: CPU Time (in seconds) consumed in the optimization.
- **Results.func**: Vector containing the best objective function value after each improvement.
- **Results.x**: Matrix containing the best vector after each improvement (in rows).
- **Results.time**: Vector containing the CPU time consumed after each improvement.
- **Results.neval**: Vector containing the number of evaluations after each improvement.
- **Results.numeval**: Total number of function evaluations.

The structures **Results**, **problem** and **opts** are saved in a file called `VNS_report.mat`.

4.2.4 Guidelines for using *VNS*

Parameter tuning in *VNS* may help increase the efficiency for a particular problem. Here is presented an explanation of the influence of each parameter and suggestions for tuning.

- **opts.use_local**: It is activated (=1) by default but it might be deactivated for a first quick run in which a feasible or a good solution is required in a short computation time, or when the problem dimension is so high that local searches involve high computational times.
- **opts.aggr**: This option uses the aggressive scheme in which the local search is only applied when the best solution has been improved within the local search. The activation of this options makes the method visit many different neighborhoods but without refining the solutions unless a very good one is found. It might be a good option to locate promising areas or to discard poor areas and constraint the search space later.
- **opts.local_search_type**: There are two types of local searches implemented in the method. One is the first improvement, which perturbs all the decision variables in a random order, changing one unit per dimension and stopping when the intial solution has been outperformed even if there are variables still not perturbed. The second option is a best improvement, which perturbs all the decision variables and then chooses the best solution among the perturbed solution to replace (or not) the initial solution. The best improvement produces a more exhaustive search than the first improvement, but it may be highly time consuming for large-scale problems. The first improvement scheme allows a more dynamic search but can miss refined high quality solutions. In both cases the *go-beyond* principle is applied: If a solution has been improved by perturbing one dimension, we repeat the perturbation following the same direction since it is considered as a promising search direction.

- **opts.decomp**: Performing a local search over all the decision variables might be computationally inefficient in the case of large-scale problems. The Variable Neighbourhood Decomposition Search (VNDS, Hansen et al. 2001) decomposes the problem into a smaller sized one by just performing the local search over the perturbed decision variables instead of all of them. The number of perturbed decision variables is determined by the problem dimension and by the options **opts.maxdist** (see below).
- **opts.maxdist**: This option chooses the percentage (between 0 and 1), of decision variables which are perturbed simultaneously in the search. In other words, it controls the distance between the current solution and the furthest neighborhood to explore. A high percentage (e.g., 100% of the variables) produces a more exhaustive search but is more time consuming. It has been empirically proven that for many instances a low percentage of perturbed variables is efficient to locate global solutions.

4.3 Application example

To illustrate the use of *VNS* we choose the 10 dimensional Rosenbrock function with integer decision variables as an example. The code of the Rosenbrock function is available inside the installation directory of the *MEIGO* package, under the examples folder.

```
nvar=10;
problem.x_L=-5*ones(1,nvar);
problem.x_U=5*ones(1,nvar);
problem.f='rosen10';

opts.maxeval=1000;
algorithm='VNS';

[Results]=MEIGO(problem,opts,algorithm);
```

5 Parallel computation in *MEIGO*

MEIGO allows the user to exploit multi core computation with either of the implemented methods by means of their corresponding parallelizable versions: *CeSS* and *CeVNS*. The different threads for calculation are managed with *jPar* (Karbowski et al., 2008), a tool for parallelizing Matlab calculations.

5.1 *jPar* installation

In order to make your OS aware of the location of certain binaries, some folders need to be added to the OS path during the installation. A guide on how to do this, can be found in:

`http://www.java.com/es/download/help/path.xml`

1. Download *jPar* from `http://www.mathworks.com/matlabcentral/fileexchange/24924` and copy it in your computer, for example in the directory `$MATLAB_ROOT/toolbox`.
2. JDK (Java Development Kit) is required to be installed in order to build *jPar*; since some users have reported problems with JDK 1.7, it is advised to use JDK 1.6.x. If you don't have JDK 1.6 installed, please install it. Check if you have the correct compiler version and if it is in the OS path by running:

   javac -version

In case the java compiler is not found, you will need to add the JDK folder the OS the path. Check `http://www.java.com/es/download/help/path.xml` for more information on how to do this. In case the JDK version is not the correct one, you need to install JDK 1.6 or to change the OS path order so that the correct *javac* is chosen preferentially.

3. Move to jPar directory and compile it by executing the compile.sh or compile.bat script.

4. Add a line to the file containing the route of the Java classes with the location of the *jpar* java executable. Typically you should add:
`$matlabroot/toolbox/jpar/jpar.jar`
in
`$MATLAB_ROOT/toolbox/local/classpath.txt;`

5. Overwrite the file:
`$MATLAB_ROOT/sys/java/jre/<system_arch>/jre/lib/security/java.policy`
with the file java.policy contained in the jpar folder. This is necessary to allow Java to open connections to the RMI communication server. The < *system_arch* >, depending on the machine in question, can be: glnx86, glnxa64, win32, win64, maci, etc.

6. Add the *jPar* folder to the OS path.

   *I*f possible, save the *jPar* directory into the default Matlab path. More information available at: `http://www.mathworks.es/es/help/matlab/ref/savepath.html`

5.2 Use of *jPar*

In order to run *jPar* follow these steps:

1. Add the *jPar* path to the Matlab session using:
`>> addpath(genpath('jpar_path'));`
where `jpar_path` points to the *jPar* folder.

2. Make sure there are no *jPar* solvers still running from a previous session. If there were any, stop them. Additionally, check if there are any *jPar* jobs running and kill them if there are any.

3. Start the *jPar* server by typing "jpar_server.bat start" in the DOS command prompt (Windows) or "sh ./jpar_server.sh start" (Unix), from the jPar directory.

4. Start the number of *jPar* solvers that are going to be used (1 solver = 1 thread). Each solver has to be started from a Matlab session in the *jPar* directory using:
`>> jpar_solver(['hostname']);`
where 'hostname' is the name of the host where *jPar* server is running.
To kill solvers use the command: `>> jpar_client('kill');`
To see free solvers use the command: `>> jpar_client('hosts');`.

Important: please make sure that *jPar* has been installed properly in your system before trying to use CeSS or CVNS

5.3 Quick start: How to carry out an optimization with *CeSS* or *CeVNS*

1. Install and learn how to use *jPar*. More details about *jPar* can be found in the previous section.

2. Open $n + 1$ Matlab sessions, where n is the number of parallel tasks you need to run.

3. In each of the $n+1$ Matlab sessions add *MEIGO* to the path by running the `install_MEIGO.m` script. You might also consider saving the *MEIGO* directory to the default Matlab path.

4. With exception to one of the $n+1$ opened Matlab sessions introduce the command: `jpar_solver('HOSTNAME')`.

   where HOSTNAME is the name/address where the *jPar* solver is running; if left empty default is 'localhost'.

5. Configure your problem and options. Use `get_Cess_options` or `get_CeVNS_options` in order to obtain a valid structure array for this purpose. Each element in this array contains the options and problem definitions for each parallel thread. Note that you may create your own scripts for this purpose. **All scripts must be modified before launching jPar**. Posterior modifications will not be detected by `jPar`.

6. In the remaining Matlab sessions where a *jPar* solver was not started, call *CeSS* or *CeVNS* providing the structure array with the options and problem definition, the number of iterations and a flag specifying if *jPar* will run in parallel mode or not. Straightforward examples on how to use CeSS or CeVNS are provided in sections 5.5 and 5.6, respectively.

7. After finishing the optimization, kill the *jPar* solvers and stop the *jPar* server. If the *jPar* configuration does not allow to stop the server through MATLAB, you can do it manually by shutting down MATLAB and killing the command window where *jPar* is running.

5.4 Options and problem definition

The corresponding cooperative method for either of the algorithms included in *MEIGO* (i.e., *CeSS* and *CeVNS*), is invoked using the same declaration and options (see sections 3.2 and 4.2). Here, the difference is that you must specify the problem definition and options for each cooperative thread. When defining the algorithm options (`opts`) we recommend the use of different settings for each thread of *eSS* or *VNS*. As for the problem definition, typically the same problem will be used in every thread. For example, if we want to use *CeSS* or *CeVNS* with 2 different threads you should provide the following structure array:

```
par_struct(1).problem=problem;
par_struct(1).opts=opts_thread_1;
par_struct(2).problem=problem;
par_struct(2).opts=opts_thread_2;
```

Additionally, two extra arguments must be defined:

- **n_iter**: Number of cooperative iterations to perform. Notice that if you only run 1 iteration there will be no exchange of information between threads (your are basically running a multi-start *VNS*/*eSS*).
- **is_parallel**: Defines if *jpar* will be used or not (true or false). This options is specially useful to debug your code, since with *jpar* you cannot use breakpoints. Additionally if there are errors in your objective function *jpar* will get stuck without returning any warnings.

5.4.1 Output

The *CeSS* /*CeVNS* output is a structure containing the following fields:

- **f_mean**: Contains the mean value of the objective function at each iteration.
- **fbest**: Contains the lowest value found by the objective function at each iteration.

- **iteration_res**: Contains the results returned by each *CeSS* / *VNS* thread at the end of an iteration. Check sections 3.2.5 and 4.2.3 for more information.
- **numeval**: The number of evaluations at the end of each iteration.
- **time**: The computation time at the end of each iteration.

5.5 *CeSS* application example

CeSS is a Matlab implementation of the Cooperative enhanced Scatter Search method which is part of the *MEIGO* toolbox for global optimization in bioinformatics. The *CeSS* strategy (Villaverde et al., 2012) is a general purpose technique for global optimization, suited for a computing environment with several processors. Its key feature is the cooperation between the different programs ("threads") that run in parallel in different processors. Each thread runs the enhanced Scatter Search metaheuristic (*eSS*) (Egea et al., 2009, 2010), which is also available in *MEIGO*.

To illustrate the use of *CeSS* we choose the D/m-group Shifted Schwefel's Problem 1.2 (f17) from the LSGO benchmarks (Tang et al., 2012) as an example. The Matlab code of f17 (`f17.m`) is available inside the installation directory of the *MEIGO* package. The Matlab scripts are provided with appropriate default options for testing *CeSS* with f17.

You can find an implementation of this problem in the script `run_CeSS.m` inside the examples folder of *MEIGO* toolbox. Notice that you will still need to follow the steps detailed in section 5.3. This example begins by defining the basic settings of the optimization:

```
% Optimization settings:
nthreads             = 2;    % number of threads
n_iter               = 2;    % number of cooperative iterations
is_parallel          = true; % parallel (true) or sequential (false)
maxtime_per_iteration = 50;   % time limit for each iteration
```

Then the information about number of parameters and their bounds is given:

```
% Number of parameters of the function and their bounds:
npars  = 1000;
x_L    = -100*ones(1,npars);
x_U    = 100*ones(1,npars);
```

After that, the function `get_CeSS_options.m` is called, which contains default settings for each thread:

```
% Read default optimization settings:
par_struct = get_CeSS_options(nthreads,npars,maxtime_per_iteration);
```

These settings may be overwritten in the main file:

```
% Overwrite the following default options in par_struct:
for i=1:nthreads
par_struct(i).problem.f         = 'f17'; % name of the function to be optimized
par_struct(i).problem.x_L       = x_L;   % lower parameter bounds
par_struct(i).problem.x_U       = x_U;   % upper parameter bounds
par_struct(i).opts.local.solver = 0;     % Don't use local solver for tests
par_struct(i).opts.local.finish = 0;     % Don't use local solver for tests
end
```

Finally, the CeSS algorithm is called and its results are plotted:

```
% Run CeSS:
Results = CeSS(par_struct,n_iter,is_parallel)

% Plot results:
plot(Results.time,log10(Results.f))
```

We encourage the modification of the default settings depending on your particular problem. It is important that each thread exploits different facets of the algorithms. Some promoting a more aggressive exploitation with more frequent local searches (e.g. lower `opts.local.n2`) and others which put more emphasis in global exploration.

5.6 *CeVNS* application example

CeVNS is an extension of *VNS* which makes use of parallel computation packages available in Matlab to reduce the time needed to solve a given integer programming problem (IP). This implementation builds on the ideas explored by (Villaverde et al., 2012) which showed (in a nonlinear programming context) that, at least for a set of benchmarks, cooperative instances of an optimization algorithm exchanging information from time to time produced better results than running same instances in an independent fashion with an equivalent computational cost.

Next we show an example script that illustrates how to configure the structure of options used by *CeVNS*. This example calls two CPUs to solve the integer Rosenbrock problem. It can be found in the script `run_rosen10_CeVNS.m` inside the examples folder in the *MEIGO* package. The mechanism of defining the options and problem for each thread is very similar to what was shown in the previous section for *CeSS*. Notice that you will still need to follow the steps detailed in section 5.3 before running this example.

```
% Optimization settings:
nthreads    = 2;           % number of threads
n_iter      = 2;           % number of cooperative iterations
is_parallel = true;        % parallel (true) or sequential (false)
maxtime_per_iteration = 10; % time limit for each iteration

% Number of parameters; bounds; and initial point:
npars = 50;
x_L   =-5*ones(1,npars);
x_U   = 5*ones(1,npars);
x_0   = round(rand(1,npars).*(x_U-x_L+1)+x_L-0.5);

% Read default optimization settings:
par_struct=get_CeVNS_options(nthreads,npars,maxtime_per_iteration);

% Overwrite the following default options in par_struct:
for i=1:nthreads
par_struct(i).problem.f   = 'rosen10';
par_struct(i).problem.x_L = x_L;
par_struct(i).problem.x_U = x_U;
par_struct(i).problem.x_0 = x_0;
end

% Run CeVNS:
Results = CeVNS(par_struct,n_iter,is_parallel)
```

As in the case of *CeSS* your are encouraged to provide different options for each thread of *CeVNS*. Although we provide a default set of options, we recommend that you tweak these settings for your particular problem.

Appendix A: List of options for *eSS*

Option	Description	Type	Options	Default value
User options				
opts.maxeval	Maximum number of function evaluations	Integer	–	1000
opts.maxtime	Maximum CPU time in seconds	Real [Positive]	–	60
opts.iterprint	Print information on the screen after each iteration	Binary	[0|1]	1
opts.weight	Weight for penalizing infeasible solutions	Real [Positive]	–	10^6
opts.log_var	Indexes of “logarithmic” variables	Integer [vector positive]	–	numeric(0)
opts.tolc	Tolerance for local search and constraints violation	Real [Positive]	–	10^{-5}
opts.prob_bound	Probability (0-1) of biasing the search toward the bounds	Real [Positive]	–	0.5
opts.inter_save	Saves results in a .mat file in intermediate iterations	Binary	[0|1]	0
Global options				
opts.dim_refset	Number of elements in *RefSet*	Integer ≥ 6	–	“auto”
opts.ndiverse	Number initial diverse solutions	Integer [Positive]	–	$10 \cdot nvar$
opts.combination	Type of combination of *RefSet* elements	Integer	[1|2]	1
Local options				
opts.local.solver	Local solver	String	see section 3.2.4	0
opts.local.tol	Level of tolerance in local search	Integer	[1|2|3]	2
opts.local.iterprint	Print each iteration of local solver on screen	Binary	[0|1]	0
opts.local.n1	Number of iterations before the first local search	Integer [Positive]	–	1
opts.local.n2	Number of iterations between two local searches	Integer [Positive]	–	10
opts.local.finish	Local solver for final search	String	see section 3.2.4	opts.local.solver
opts.local.bestx	Applies the local search only when *fbest* is improved	Binary	[0|1]	0
opts.local.balance	Balances between quality (= 0) and diversity (= 1)	Real [Positive]	–	0.5

Appendix B: List of options for *VNS*

Option	Description	Type	Options	Default value
opts.maxeval	Maximum number of function evaluations	Integer	–	1000
opts.maxtime	Maximum CPU time in seconds	Real [Positive]	–	60
opts.maxdist	Option related with the maximum number of perturbed variables	Real	[0—1]	0.5
opts.use_local	Use local search or not	Binary	[0—1]	1
opts.aggr	Applies aggressive search	Binary	[0—1]	0
opts.local_search_type	Applies first (1) or best (2) improvement in the local search	Integer	[1—2]	1
opts.decomp	Decomposes the local search	Binary	[0—1]	1

References

de la Maza, M. and Yuret, D. (1994). Dynamic hill climbing. *AI Expert*, 9(3):26–31.

Dennis, J. E., Gay, D. M., and Welsch, R. E. (1981). An adaptive non-linear least-squares algorithm. *ACM Transactions on Mathematical Software*, 7(3):348–368.

Egea, J., Balsa-Canto, E., García, M.-S., and Banga, J. (2009). Dynamic optimization of nonlinear processes with an enhanced scatter search method. *Industrial & Engineering Chemistry Research*, 48(9):4388–4401.

Egea, J., Martí, R., and Banga, J. (2010). An evolutionary algorithm for complex process optimization. *Computers & Operations Research*, 37(2):315–324.

Hansen, P., Mladenović, N., and Pérez-Brito, D. (2001). Variable neighborhood decomposition search. *Journal of Heuristics*, 7(4):335–350.

Hooke, R. and Jeeves, T. (1961). Direct search solution of numerical and statistical problems. *Journal of the ACM*, 8(2):212–229.

Karbowski, A., Majchrowski, M., and Trojanek, P. (2008). jpar–a simple, free and lightweight tool for parallelizing matlab calculations on multicores and in clusters. *9th International Workshop on State-of-the-Art in Scientific and Parallel Computing.*

Mladenović, N. and Hansen, P. (1997). Variable neighborhood search. *Computers and Operations Research*, 24:1097–1100.

Mladenović, N. and Hansen, P. (2010). Variable neighbourhood search: methods and applications. *Annals of Operations Research*, 175(1):367–407.

Rodríguez-Fernández, M., Egea, J. A., and Banga, J. R. (2006). Novel metaheuristic for parameter estimation in nonlinear dynamic biological systems. *BMC Bioinformatics*, 7:483+.

Tang, K., Yang, Z., and Weise, T. (2012). Competition on large scale global optimization. *IEEE World Congress on Computational Intelligence*, `http://staff.ustc.edu.cn/~ketang/cec2012/lsgo_competition.htm`.

The MathWorks™ (2008). Optimization toolbox 4 user's guide.

Villaverde, A. F., Egea, J. A., and Banga, J. R. (2012). A cooperative strategy for parameter estimation in large scale systems biology models. *BMC Systems Biology*, 6:75.

Wächter, A. and Biegler, L. (2006). On the implementation of an interior point filter line search algorithm for large-scale nonlinear programming. *Mathematical Programming*, 106(1):25–57.

Ye, Y. (1987). *Interior algorithms for linear, quadratic and linearly constrained non-linear programming.* PhD thesis, Stanford University.
