# Supplementary material for: MEIGO: an open-source software suite based on metaheuristics for global optimization in systems biology and bioinformatics
Source: BMC Bioinformatics. 2014 May 10;15:136. doi: 10.1186/1471-2105-15-136 (PMC4025564; doi:10.1186/1471-2105-15-136)
Supplement: Additional file 2 — MEIGO - R’s user’s manual. The file includes the users manual of the R version of MEIGO. [file 1471-2105-15-136-S2.pdf]

MEIGOR: a software suite based on metaheuristics for global optimization in systems biology and bioinformatics

Jose A. Egea*[3], David Henriques[1], Thomas Cokelaer[2], Alejandro F. Villaverde[2], Aidan MacNamara[2], Diana-Patricia Danciu[2], Julio R. Banga †[2], and Julio Saez-Rodriguez ‡[2]

[1]Instituto de Investigaciones Marinas-CSIC, Vigo, Spain
[2]European Bioinformatics Institute, Saez-Rodriguez group, Cambridge, United Kingdom
[3]Universidad Politécnica de Cartagena, Cartagena, Spain

February 15, 2014

Contents

1 Introduction **2**

2 Installing *MEIGO* **2**

3 Continuous and mixed-integer problems: Enhanced Scatter Search (*eSSR*) **3**
3.1 Quick start: How to carry out an optimization with *eSSR* . . . 3
3.2 *eSSR* usage . . . 4
3.2.1 Problem definition . . . 4
3.2.2 User options . . . 5
3.2.3 Global options . . . 5
3.2.4 Local options . . . 5
3.2.5 Output . . . 6
3.2.6 Guidelines for using *eSSR* . . . 7
3.2.7 Extra tool: *essR_multistart* . . . 8
3.3 Examples . . . 8
3.3.1 Unconstrained problem . . . 8
3.3.2 Constrained problem . . . 9
3.3.3 Constrained problem with equality constraints . . . 10
3.3.4 Mixed integer problem . . . 11
3.3.5 Dynamic parameter estimation problem using *NL2SOL* . . . 12
3.3.6 *essR_multistart* application . . . 14

4 Integer optimization: Variable Neighbourhood Search (*VNSR*) **14**
4.1 Quick start: How to carry out an optimization with *VNSR* . . . 15
4.2 *VNSR* usage . . . 15
4.2.1 Problem definition . . . 15

*josea.egea@upct.es
†julio@iim.csic.es
‡saezrodriguez@ebi.ac.uk

4.2.2 *VNSR* options . . . 15
4.2.3 Output . . . 16
4.3 Guidelines for using *VNSR* . . . 16
4.4 Application example . . . 17

5 Bayesian inference: BayesFit **18**
5.1 Quick Start . . . 18
5.2 Quick Start: How to define posterior distributions of parameters with *BayesFit* . . . 18

6 Parallel computation in *MEIGO* **19**
6.1 Quick notes about the parallel computing packages . . . 19
6.1.1 Snowfall . . . 19
6.2 Usage . . . 20
6.2.1 Options . . . 20
6.2.2 Output . . . 21
6.3 *CeSSR* application example . . . 21
6.4 *CeVNSR* application example . . . 22

7 Applications from Systems Biology **23**
7.1 Using eSS to fit a dynamic model . . . 23
7.2 Accelerating the computations with CeSSR . . . 29
7.3 Using VNS to optimize logic models . . . 31
7.4 Using CeVNSR to accelerate computations . . . 36
7.5 Using BayesFit to optimize logic models . . . 38

1 Introduction

MEIGO is an optimization suite programmed in R which implements metaheuristics for solving different nonlinear optimization problems in both continuous and integer domains arising in systems biology, bioinformatics and other areas. It consists of two main metaheuristics: the enhanced scatter search method, *eSSR* [?, ?] for continuous and mixed-integer problems, and the variable neighbourhood search metaheuristic [?], for integer problems.

”A metaheuristic is an iterative master procedure that guides and modifies the operations of subordinate heuristics to efficiently produce high quality solutions” [?] .

Metaheuristics are useful for solving problems that due to its complexity cannot be solved by deterministic global optimization solvers or where the usage of a local solver systematically converges to a local solutions. Metaheuristics do not guarantee optimality but are usually efficient in locating the vicinity of the global solution in modest computational time.

Both *eSS* and *VNS* are hybrid solvers. This means that the stochastic optimization methods are combined with local solvers to improve the efficiency.

Both metaheuristics have been implemented in R and can be invoked within the *MEIGO* framework. Additionally, MEIGO allows for Bayesian inference of parameters using the method BayesFit. This manual describes all methods, their corresponding parallelizable versions (in the case of *eSS* and *VNS*), and guides the user to implement his/her own optimization problems and to choose the best set of options for a particular instance.

2 Installing *MEIGO*

Before installing MEIGOR we will need to install two additional packages:

```
install.packages("Rsolnp")
install.packages("snowfall")
```

Install MEIGOR:

```
install.packages("MEIGOR_0.99.6.tar.gz",type="source")
```

3 Continuous and mixed-integer problems: Enhanced Scatter Search (*eSSR*)

eSSR is an R implementation of the enhanced scatter search method [?, ?] which is part of the *MEIGO* toolbox for global optimization in bioinformatics. It is a metaheuristic which seeks the global minimum of mixed-integer nonlinear programming (MINLP) problems specified by

$$\min_x f(x, p_1, p_2, ..., p_n)$$

subject to

$$\begin{aligned} c_{eq} &= 0 \\ c_L \le c(x) &\le c_U \\ x_L \le x &\le x_U \end{aligned}$$

where x is the vector of decision variables, and x_L and x_U its respective bounds. $p_1, \ldots, p_n$ are optional extra input parameters to be passed to the objective function (see examples in sections 3.3.3, 3.3.5). c_{eq} is a set of equality constraints. $c(x)$ is a set of inequality constraints with lower and upper bounds, c_L and c_U. Finally, $f(x, p_1, p_2, ..., p_n)$ is the objective function to be minimized.

3.1 Quick start: How to carry out an optimization with *eSSR*

```
  library(MEIGOR)
problem<-list(f=objective, x_L=rep(-1,2), x_U=rep(1,2))
opts<-list(maxeval=500, local_solver='DHC')
```

- Type: `Results<-MEIGO(problem,opts,algorithm="ESS")` . If your problem has additional constant parameters to be passed to the objective function, they are declared as input parameters after "opts'" (e.g., type `Results<-MEIGO(problem,opts,algorithm="ESS",p1,p2)` if your model has two extra input parameters, p_1 and p_2).

Regarding the objective function, the input parameter is the decision vector (with extra parameters $p_1, p_2, \ldots, p_n$ if they were defined before calling the solver). The objective function must provide a scalar output parameter (the objective function value) and, for constrained problems, a second output parameter, which is a vector containing the values of the constraints. For problems containing equality constraints (= 0), they must be defined before the inequality constraints. Some examples are provided in section 3.3. For a quick reference, consider the following example which will be later extended in section 3.3.3.

$$\min_x f(x) = -x_4$$

subject to

$$
\begin{aligned}
x_4 - x_3 + x_2 - x_1 + k_4 x_4 x_6 &= 0 \\
x_1 - 1 + k_1 x_1 x_5 &= 0 \\
x_2 - x_1 + k_2 x_2 x_6 &= 0 \\
x_3 + x_1 - 1 + k_3 x_3 x_5 &= 0 \\
x_5^{0.5} + x_6^{0.5} &\leq 4 \\
0 \leq x_1, x_2, x_3, x_4 &\leq 1 \\
0 \leq x_5, x_6 &\leq 16
\end{aligned}
$$

with k_1, k_2, k_3, k_4 being extra parameters defined before calling the solver. The objective function for this problem would be:

```
ex3<-function(x,k1,k2,k3,k4){
 f=-x[4];
 #Equality constraints, declare these before the inequality ones
 g<-rep(0,5);
 g[1]=x[4]-x[3]+x[2]-x[1]+k4*x[4]*x[6];
 g[2]=x[1]-1+k1*x[1]*x[5];
 g[3]=x[2]-x[1]+k2*x[2]*x[6];
 g[4]=x[3]+x[1]-1+k3*x[3]*x[5];
 #inequality constraints
 g[5]=x[5]^0.5+x[6]^0.5
 return(list(f=f,g=g));
 }
```

This objective function can be invoked like this (let us assume that x has dimension 6 and we define the 4 extra input parameter k_1 to k_4).

`ex3(c(1,1,1,1,1,1),0.2,0.5,-0.1,0.9)`

3.2 *eSSR* usage

3.2.1 Problem definition

In order to solve an optimization problem with *eSSR*, a list (named **problem** here) containing the following fields must be defined:

- **f**: String containing the name of the objective function.
- **x_L**: Vector containing the lower bounds of the variables.
- **x_U**: Vector containing the upper bounds of the variables.

Besides, there are two optional fields

- **x_0**: Vector or matrix containing the given initial point(s).
- **f_0**: Function values of the initial point(s). These values MUST correspond to feasible points.
- **vtr**: Objective function value to be reached.

If the problem contains additional constraints and/or integer or binary variables, the following fields should also be defined:

- **neq**[1]: Number of equality (= 0) constraints.
- **c_L**: Vector defining the lower bounds of the inequality constraints.
- **c_U**: Vector defining the upper bounds of the inequality constraints.
- **int_var**[2]: Number of integer variables.
- **bin_var**[2]: Number of binary variables.

3.2.2 User options

The user may define a set of different options related to the optimization problem. They are defined in another list (named **opts** here) which has the following fields:

- **maxeval**: Maximum number of function evaluations (default: 1000).
- **maxtime**: Maximum CPU time in seconds (default: 60).
- **iterprint**: Print information on the screen after each iteration. 0: Deactivated; 1: Activated (default: 1).
- **weight**: Weight that multiplies the penalty term added to the objective function in constrained problems (default: 10^6).
- **log_var**: Indexes of the variables which will be analyzed using a logarithmic distribution instead of an uniform one[3] (default: numeric(0)). See an example in Section 3.3.5.
- **tolc**: Maximum constraint violation allowed. This is also used as a tolerance for the local search (default: 10^{-5}).
- **prob_bound**: Probability (0-1) of biasing the search towards the bounds. 0: Never bias to bounds; 1: Always bias to bounds (default: 0.5).
- **inter_save**: Saves results of intermediate iterations in **eSSR_report.Rdata**. Useful for very long runs. 0: deactivated; 1: activated (default: 0).

3.2.3 Global options

A set of options related to the global search phase of the algorithm may also be defined also within the list **opts**:

- **dim_refset**: Number of elements d in *RefSet* (default: *"auto"*, $\frac{d^2-d}{10 \cdot nvar} \geq 0$).
- **ndiverse**: Number of solutions generated by the diversificator in the initial stage (default: *"auto"*, $10 \cdot nvar$).
- **combination**: Type of combination of *RefSet* elements. 1: Hyper-rectangles combinations; 2: Linear combinations (default: 1).

[1]In problems with equality constraints they must be declared first before inequality constraints (See example 3.3.3)
[2]For mixed integer problems, the variables must be defined in the following order: [cont., int., bin.].
[3]Useful when the bounds of a decision variables have different orders of magnitude and they are both positive.

3.2.4 Local options

eSSR is a global optimization method which performs local searches from selected initial points to accelerate the convergence to optimal solutions. Some options regarding the local search can be defined in the **opts** list, as follows:

- **local_solver**: Local solver to perform the local search. Different solvers available and their names must be introduced as strings. Please note that at the moment no local solver for mixed-integer problems is available within *eSSR*:
  - *NM*:[3]. Nelder and Mead method for unconstrained problems [?].
  - *BFGS*:[3] Quasi-Newton method for unconstrained problems [?].
  - *CG*:[3] Conjugate gradients method for unconstrained problems [?].
  - *LBFGSB*:[3] Quasi-Newton method which allows box constraints [?].
  - *SA*:[3] Variant of simulated annealing for unconstrained problems [?].
  - *SOLNP*: the SQP method [4] by [?].
  - *DHC*: Direct search method [?].
  - *NL2SOL*: Specific method for non-linear least squares problems [?], implemented through the *nls* R function.
- **local_tol**: Level of tolerance in local search. 1: Relaxed; 2: Medium; 3: Tight (default: 2 in intermediate searches and 3 in the final stage).
- **local_iterprint**: Print each iteration of local solver on screen (only for local solvers that allow it). 0: Deactivated; 1: Activated (default: 0).
- **local_n1**: Number of iterations before applying the local search for the first time (default: 1).
- **local_n2**: Number of function iterations in the global phase between two consecutive local searches (default: 10).
- **local_finish**: Chooses the local solver to apply a final refinement to the best solution found once the optimization is finished (default: same as **local_solver**).
- **local_bestx**: If activated (i.e., positive value), applies the local search only when the algorithm finds a solution better than the current best solution. 0: Deactivated; 1: Activated (default: 0).
- **local_balance**: Balances between quality (=0) and diversity (=1) for choosing initial points for the local search (default 0.5).

Note that, for some problems, the local search may be inefficient, spending a high computation time to provide low quality solutions. This is the case of many noisy or ill-posed problems. In these instances, the local search may be deactivated by user by defining the value of the field **solver** as zero.

When using *NL2SOL* as local solver, the objective function value must be formulated as the square of the sum of differences between the experimental and predicted data (i.e., $\sum_{i=1}^{ndata}(yexp_i - yteor_i)^2$). Besides, a third output argument must be defined in the objective function: a vector containing those residuals (i.e., $R = [(yexp_1 - yteor_1), (yexp_2 - yteor_2), \ldots, (yexp_{ndata} - yteor_{ndata})]$). In Section 3.3.5 an application example illustrates the use of this local method.

[3]Included in the R *optim* function. http://stat.ethz.ch/R-manual/R-devel/library/stats/html/optim.html

[4]Implemented by Alexios Ghalanos and Stefan Theussl [?]. Requires the packages **Rsolnp** and **truncnorm** http://cran.r-project.org/web/packages/Rsolnp/index.html

3.2.5 Output

eSSR's output is a list (called **Results** here) containing the following fields:

- **Results$fbest**: Best objective function value found after the optimization.
- **Results$xbest**: Vector providing the best function value found.
- **Results$cpu_time**: CPU Time (in seconds) consumed in the optimization.
- **Results$f**: Vector containing the best objective function value after each iteration.
- **Results$x**: Matrix containing the best vector after each iteration.
- **Results$time**: Vector containing the CPU time consumed after each iteration.
- **Results$neval**: Vector containing the number of evaluations after each iteration.
- **Results$numeval**: Total number of function evaluations.
- **Results$local_solutions**: Matrix of local solutions found.
- **Results$local_solutions_values**: Function values of the local solutions.
- **Results$Refset$x**: Matrix of solutions in the final *Refset* after the optimization.
- **Results$Refset$f**: Objetive function values of the final *Refset* members after the optimization.
- **Results$Refset$fpen**: Penalized objetive function values of the final *Refset* members after the optimization. The values for feasible solutions will coincide with their corresponding **Results$Refset$f** values.
- **Results$Refset$const**: Matrix containing the values of the constraints (those whose bounds are defined in **problem$c_L** and/or **problem$c_U**) for each of the solutions in the final *Refset* after the optimization.
- **Results$end_crit**: Criterion to finish the optimization:
  - 1: Maximum number of function evaluations achieved.
  - 2: Maximum allowed CPU time achieved.
  - 3: Value to reach achieved.

The list **Results** as well as **problem** and **opts** are stored and saved in a file called `eSSR_report.RData`.

3.2.6 Guidelines for using *eSSR*

Although *eSSR* default options have been chosen to be robust for a high number of problems, the tuning of some parameters may help increase the efficiency for a particular problem. Here is presented a list of suggestions for parameter choice depending on the type of problem the user has to face.

- If the problem is likely to be convex, an early local search can find the optimum in short time. For that it is recommended to set the parameter **opts$local_n1 = 1**. Besides, setting **opts$local_n2 = 1** too, the algorithm increases the local search frequency, becoming an "intelligent" multistart.
- When the bounds differ in several orders of magnitude, the decision variables indexes may be included in **log_var**.

- For problems with discontinuities and/or noise, the local search should either be deactivated or performed by a direct search method. In those cases, activating the option **opts$local_bestx = 1** may help reduce the computation time wasted in useless local searches by performing a local search only when the best solution found has been improved.
- When the function values are very high in absolute value, the weight (**opts$weight**) should be increased to be at least 3 orders of magnitude higher than the mean function value of the solutions found.
- When the search space is very big compared to the area in which the global solution may be located, a first investment in diversification may be useful. For that, a high value of **opts$ndiverse** can help finding good initial solutions to create the initial *RefSet*. A preliminary run with aggressive options can locate a set of good initial solutions for a subsequent optimization with more robust settings. This aggressive search can be performed by reducing the size of the *RefSet* (**opts$dim_refset**). A more robust search is produced increasing the *RefSet* size.
- If local searches are very time-consuming, their tolerance can be relaxed by reducing the value of **opts$local_tol** not to spend a long time in local solution refinements.
- When there are many local solutions close to the global one, the option **opts$local_balance** should be set close to 0.

3.2.7 Extra tool: *essR_multistart*

This tool allows the user to perform a multistart optimization procedure with any of the local solvers implemented in *eSSR* using the same problem declaration. *essR_multistart* can be invoked within *MEIGO* using the same structure setting the parameter `algorithm="MULTISTART"` .

```
Results_multistart<-MEIGO(problem,opts,algorithm="MULTISTART",p1,p2,...)
```

The list **problem** has the same fields as in *essR* (except **problem$vtr** which does not apply here).

The list **opts** has only a few fields compared with *essR* (i.e.,**opts$ndiverse**, **opts$local_solver**, **opts$iterprint**, **opts$local_tol** and **opts$local_iterprint**). They all work like in *essR* except **opts$ndiverse**, which indicates the number of initial points chosen for the multistart procedure. A histogram with the final solutions obtained and their frequency is presented at the end of the procedure.

The output list **Results_multistart** contains the following fields:

- **$fbest**: Best objective function value found after the multistart optimization.
- **$xbest**: Vector providing the best function value.
- **$x0**: Matrix containing the vectors used for the multistart optimization.
- **$f0**: Vector containing the objective function values of the vectors in **Results_multistart$x0**.
- **$func**: Vector containing the objective function values obtained after every local search.
- **$xxx**: Matrix containing the vectors provided by the local optimizations.
- **$no_conv**: Matrix containing the initial points that did not converge to any solution.
- **$nfuneval**: Matrix containing the number of function evaluations performed in every optimization.

The lists **problem**, **opts** and **Results_multistart** are saved in a file called `Results_multistart$RData`.

3.3 Examples

In this section we will illustrate the usage of *eSSR* within *MEIGO* for solving different instances.

3.3.1 Unconstrained problem

$$\min_x f(x) = 4x_1^2 - 2.1x_1^4 + 1/3x_1^6 + x_1x_2 - 4x_2^2 + 4x_2^4$$

subject to

$$-1 \leq x_1, x_2 \leq 1$$

The objective function is defined in `ex1.R`. Note that being an unconstrained problem, there is only one output argument, f.

```
ex1 <- function(x){
 y<-4*x[1]*x[1]-2.1*x[1]^4+1/3*x[1]^6+x[1]*x[2]-4*x[2]*x[2]+4*x[2]^4;
 return(y)
 }
```

The solver is called in `main_ex1.R`. This problem has two known global optima in $x^* = (0.0898, -0.7127)$ and $x^* = (-0.0898, 0.7127)$ with $f(x^*) = -1.03163$.
Options set:

- Maximum number of function evaluations set to 500.
- Maximum number of initial diverse solutions set to 40.
- Local solver chosen: *DHC*.
- Local solver for final refinement: *LBFGSB*.
- Show the information provided by local solvers on screen.

```
#========================
#PROBLEM SPECIFICATIONS
# ========================
problem<-list(f="ex1",x_L=rep(-1,2),x_U=rep(1,2))
opts<-list(maxeval=500, ndiverse=40, local_solver='DHC',
 local_finish='LBFGSB', local_iterprint=1)
#========================
# END OF PROBLEM SPECIFICATIONS
#========================

Results<-MEIGO(problem,opts,algorithm="ESS");
```

3.3.2 Constrained problem

$$\min_x f(x) = -x_1 - x_2$$

subject to

$$\begin{aligned} x_2 &\leq 2x_1^4 - 8x_1^3 + 8x_1^2 + 2 \\ x2 &\leq 4x_1^4 - 32x_1^3 + 88x_1^2 - 96x_1 + 36 \\ 0 &\leq x_1 \leq 3 \\ 0 &\leq x_2 \leq 4 \end{aligned}$$

The objective function is defined in ex2.R. Note that being a constrained problem, there are two output argument, f and g.

```
ex2<-function(x){
 F=-x[1]-x[2];
 g<-rep(0,2);
 g[1]<-x[2]-2*x[1]^4+8*x[1]^3-8*x[1]^2;
 g[2]<-x[2]-4*x[1]^4+32*x[1]^3-88*x[1]^2+96*x[1];
 return(list(F=F,g=g))
 }
```

The solver is called in main_ex2.R. The global optimum for this problem is located in $x^* = [2.32952, 3.17849]$ with $f(x^*) = -5.50801$.
Options set:

- Maximum number of function evaluations set to 750.
- Increase frequency of local solver calls. The first time the solver is called after 2 iterations. From that moment, the local solver will be called every 3 iterations.

```
#========================
#PROBLEM SPECIFICATIONS
#========================
problem<-list(f="ex2",x_L=rep(0,2),x_U=c(3,4),
 c_L=rep(-Inf,2), c_U=c(2,36))
opts<-list(maxeval=750, local_solver="DHC", local_n1=2, local_n2=3)
#=======================
#END OF PROBLEM SPECIFICATIONS
# =======================

Results<-MEIGO(problem,opts,algorithm="ESS");
```

3.3.3 Constrained problem with equality constraints

$$\min_x f(x) = -x_4$$

subject to

$$\begin{aligned} x_4 - x_3 + x_2 - x_1 + k_4 x_4 x_6 &= 0 \\ x_1 - 1 + k_1 x_1 x_5 &= 0 \\ x_2 - x_1 + k_2 x_2 x_6 &= 0 \\ x_3 + x_1 - 1 + k_3 x_3 x_5 &= 0 \\ x_5^{0.5} + x_6^{0.5} &\leq 4 \\ 0 \leq x_1, x_2, x_3, x_4 &\leq 1 \\ 0 \leq x_5, x_6 &\leq 16 \end{aligned}$$

with $k_1 = 0.09755988$, $k_3 = 0.0391908$ $k_2 = 0.99k_1$ and $k_4 = 0.9k_3$. The objective function is defined in ex3.R. Note that equality constraints must be declared before inequality constraints. Parameters $k_1, \ldots, k_4$ are passed to the objective function through the main script, therefore they do not have to be calculated in every function evaluation. See the input arguments below.

```
ex3<-function(x,k1,k2,k3,k4){
 f=-x[4];
 #Equality constraints
 g<-rep(0,5);
 g[1]=x[4]-x[3]+x[2]-x[1]+k4*x[4]*x[6];
 g[2]=x[1]-1+k1*x[1]*x[5];
 g[3]=x[2]-x[1]+k2*x[2]*x[6];
 g[4]=x[3]+x[1]-1+k3*x[3]*x[5];

 #Inequality constraint
 g[5]=x[5]^0.5+x[6]^0.5;
 return(list(f=f,g=g));
 }
```

The solver is called in texttttmain_ex3.R. The global optimum for this problem is located in $x^* = [0.77152, 0.516994, 0.204189, 0.$
with $f(x^*) = -0.388811$.
Options set:

- Number of equality constraints set to 4 in `problem$neq`..
- Fields `problem$c_L` and `problem$c_U` only contain bounds for inequality constraints.
- Maximum computation time set to 5 seconds.
- Local solver chosen: *solnp*. It requires the R packages *Rsonlp* and *truncnorm*.
- Parameters $k_1, \ldots, k_4$ are passed to the main routine as input arguments.

```
#=========================
#PROBLEM SPECIFICATIONS
#=========================
problem<-list(f="ex3",x_L=rep(0,6),x_U=c(rep(1,4),16,16),
 neq=4, c_L=-Inf, c_U=4)
opts<-list(maxtime=5, local_solver='solnp')
#=========================
#END OF PROBLEM SPECIFICATIONS
#=========================
k1=0.09755988;
k3=0.0391908;
k2=0.99*k1;
k4=0.9*k3;
Results<-MEIGO(problem,opts,algorithm="ESS",k1,k2,k3,k4);
```

3.3.4 Mixed integer problem

$$\min_x f(x) = x_2^2 + x_3^2 + 2x_1^2 + x_4^2 - 5x_2 - 5x_3 - 21x_1 + 7x_4$$

subject to

$$\begin{aligned} x_2^2 + x_3^2 + x_1^2 + x_4^2 + x_2 - x_3 + x_1 - x_4 &\leq 8 \\ x_2^2 + 2x_3^2 + x_1^2 + 2x_4^2 - x_2 - x_4 &\leq 10 \\ 2x_2^2 + x_3^2 + x_1^2 + 2x_2 - x_3 - x_4 &\leq 5 \\ 0 \leq x_i &\leq 10 \quad \forall i \in [1, \ldots, 4] \end{aligned}$$

Integer variables: x_2, x_3 and x_4. In the function declaration (`ex4.R`) they must have the last indexes.

```
ex4<-function(x){
 F = x[2]^2 + x[3]^2 + 2.0*x[1]^2 + x[4]^2 - 5.0*x[2] - 5.0*x[3] - 21.0*x[1] + 7.0*x[4];
 g<-rep(0,3);
 g[1] = x[2]^2 + x[3]^2 + x[1]^2 + x[4]^2 + x[2] - x[3] + x[1] - x[4];
 g[2] = x[2]^2 + 2.0*x[3]^2 + x[1]^2 + 2.0*x[4]^2 - x[2] - x[4];
 g[3] = 2.0*x[2]^2 + x[3]^2 + x[1]^2 + 2.0*x[2] - x[3] - x[4];
 return(list(F=F, g=g));
 }
```

The solver is called in `main_ex4.R`. The global optimum for this problem is located in $x^* = [2.23607, 0, 1, 0]$ with $f(x^*) = -40.9575$.
Options set:

- An initial point is specified.
- The number of integer variables is specified (mandatory).
- No local solver is available for mixed-integer problems at the moment.
- Stop criterion determined by the CPU time (2 seconds).

```
#========================= PROBLEM SPECIFICATIONS ===========================
problem<-list(f="ex4", x_L=rep(0,4), x_U=rep(10,4), x_0=c(3,4,5,1),
 int_var=3, c_L=rep(-Inf,3), c_U=c(8,10,5))
opts<-list(maxtime=2)
#========================= END OF PROBLEM SPECIFICATIONS =====================
Results<-MEIGO(problem,opts,algorithm="ESS");
```

3.3.5 Dynamic parameter estimation problem using *NL2SOL*

Here we will illustrate the use of *eSSR* within *MEIGO* using *NL2SOL* as local solver. In particular, the problem considered is the isomerization of α-pinene [?].

$$\min_p J = \sum_{j=1}^{5}\sum_{i=1}^{8}(y_j(p,t_i) - \tilde{y}_{ji})^2$$

subject to the system dynamics

$$\begin{aligned}
\frac{dy_1}{dt} &= -(p_1+p_2)y_1 \\
\frac{dy_2}{dt} &= p_1 y_1 \\
\frac{dy_3}{dt} &= p_2 y_1 - (p_3+p_4)y_3 + p_5 y_5 \\
\frac{dy_4}{dt} &= p_3 y_3 \\
\frac{dy_5}{dt} &= p_4 y_3 - p_5 y_5
\end{aligned}$$

and subject to parameter bounds

$$0 \le x_i \le 1 \quad \forall i \in [1,\dots,5]$$

In order to use *NL2SOL* as local solver, in the script `ex5.R` there must be three output arguments: apart from the objective function and the constraints (empty in this case), a vector R containing the squares of the residuals must be defined. Please note that the library *deSolve* must be loaded in order to use the ODE integrator *lsodes*.

```
ex5<-function(x,texp,yexp){
 yini<-c(100, 0, 0, 0 ,0);
 times<-texp;
 out   <- lsodes(yini, times, ex5_dynamics, parms = x)

 tout<-out[,1];
 yout<-out[,-1];
 J <- sum((yout-yexp)^2);
 g<-0;
 residuals<-(yout-yexp);

 return(list(J,g,residuals))
 }
#**************************************************
#Function of the dynamic system
ex5_dynamics<-function(t,y,p){
 dy<-rep(0,5)

 dy[1]<--(p[1]+p[2])*y[1];
 dy[2]<-p[1]*y[1];
 dy[3]<-p[2]*y[1]-(p[3]+p[4])*y[3]+p[5]*y[5];
 dy[4]<-p[3]*y[3];
 dy[5]<-p[4]*y[3]-p[5]*y[5];
 return(list(dy))
 }
#**************************************************
```

The solver is called in `main_ex5.R`. The global optimum for this problem is located in $\mathbf{p}^* = [5.93 \cdot 10^{-5}, 2.96 \cdot 10^{-5}, 2.0 \cdot 10^{-5}, 2.75 \cdot 10^{-4}, 4.00 \cdot 10^{-5}]$, with $f(\mathbf{p}^*) = 19.88$.
Options set:

- An initial point is specified.
- Maximum number of evaluations is 1000.
- All the variables are declared as *log_var*.
- We choose the *NL2SOL* local solver.
- Save results in a file after every iteration.

```
#======================
#PROBLEM SPECIFICATIONS
#======================
problem<-list(f="ex5", x_L=rep(0,5), x_U=rep(1,5), x_0=rep(0.5,5))
opts<-list(maxeval=1000, log_var=1:5,
 local_solver='NL2SOL', inter_save=1)
#=======================
```

```
#END OF PROBLEM SPECIFICATIONS
#======================
#time intervals

texp<-c(0, 1230, 3060, 4920, 7800,
 10680, 15030, 22620, 36420)
#Distribution of species concentration
#             y(1)    y(2)    y(3)    y(4)    y(5)

yexp<-rbind(c(100.0, 0.0, 0.0, 0.0, 0.0),
 c(88.35, 7.3, 2.3, 0.4, 1.75),
 c( 76.4  , 15.6,    4.5 ,   0.7 ,   2.8),
 c(65.1 ,   23.1  ,   5.3 ,    1.1 ,    5.8),
 c(50.4  ,  32.9   ,  6.0   ,  1.5   ,  9.3),
 c(37.5  ,  42.7 ,    6.0 ,    1.9  ,  12.0),
 c(25.9  ,  49.1  ,   5.9  ,   2.2 ,   17.0),
 c( 14.0  ,  57.4    , 5.1  ,   2.6   , 21.0),
 c(4.5 ,   63.1  ,   3.8   ,  2.9  ,  25.7));
Results<-MEIGO(problem,opts,algorithm="ESS",texp,yexp);
```

3.3.6 *essR_multistart* application

An application of *essR_multistart* within *MEIGO* on the problem *ex3* using *solnp* as local solver is presented in the script `main_multistart_ex3.R`. The number of initial points chosen is 25.

```
#========================
#PROBLEM SPECIFICATIONS
#========================
problem<-list(f="ex3",x_L=rep(0,6),x_U=c(rep(1,4),16,16),
 neq=4, c_L=-Inf, c_U=4)
opts<-list(ndiverse=25, local_solver='SOLNP', local_tol=3)
#========================
#END OF PROBLEM SPECIFICATIONS
#========================
k1=0.09755988;
k3=0.0391908;
k2=0.99*k1;
k4=0.9*k3;
Results<-MEIGO(problem,opts,algorithm="multistart",k1,k2,k3,k4);
```

4 Integer optimization: Variable Neighbourhood Search (*VNSR*)

VNSR is an R implementation of the Variable Neighbourhood Search (VNS) metaheuristic which is part of the *MEIGO* toolbox for global optimization in bioinformatics. VNS was first proposed by [**?**] for solving combinatorial and/or global optimization problems. The method guides a trial solution to search for an optimum in a certain area. After this optimum is located, the trial solution is perturbed to start searching in a new area (or neighbourhood). New neighbourhoods are defined following a distance criterion in order to achieve a good diversity in the search. Different variants of the method have been published in recent years in order to adapt it to different types of problems [**?**]. VNSR implements some of this variants by means of different tunning parameters.

VNSR seeks the global minimum of integer programming (IP) problems specified by

$$\min_{x} f(x, p_1, p_2, ..., p_n)$$

subject to

$$x_L \le x \le x_U$$

where x is the vector of (integer) decision variables, and x_L and x_U its respective bounds. $p_1, \ldots, p_n$ are optional extra input parameters to be passed to the objective function, f, to be minimized. The current *VNSR* version does not handle constraints apart from bound constrained, so that the user should formulate his/her own method (i.e., a penalty function) to solve constrained problems.

4.1 Quick start: How to carry out an optimization with *VNSR*

- Define your problem and options (see the following sections)
- Type: `Results<-MEIGO(problem,opts, algorithm="VNS")`. If your problem has additional constant parameters to be passed to the objective function, they are declared as input parameters after "opts" (e.g., type `Results<-MEIGO(problem,opts,algortim="VNS'",p1,p2)` if your model has two extra input parameters, p_1 and p_2).

Regarding the objective function, the input parameter is the decision vector (with extra parameters $p_1, p_2, \ldots, p_n$ if they were defined before calling the solver). The objective function must provide a scalar output parameter (the objective function value).

4.2 *VNSR* usage

4.2.1 Problem definition

A list named **problem** containing the following fields must be defined for running *VNSR*:

- **f**: String containing the name of the objective function.
- **x_L**: Vector containing the lower bounds of the variables.
- **x_U**: Vector containing the upper bounds of the variables.
- **x_0**: Vector containing the initial solution to start the search. If this field is not defined then the algorithm will choose an initial point randomly chosen within the bounds.

4.2.2 *VNSR* options

The user may define a set of different options related to the integer optimization problem. They are defined in another list (named **opts** here) which has the following fields.

- **maxeval**: Maximum number of function evaluations (default: 1000).
- **maxtime**: Maximum CPU time in seconds (default: 60).
- **maxdist**: Percentage of the problem dimension which will be perturbed in the furthest neighborhood (vary between 0-1, default: 0.5).

- **use_local**: Uses local search (1) or not (0). Default:1.

The following options only apply when the local search is activated (i.e., opts$use_local=1)

- **aggr**: Aggressive search. The local search is only applied when the best solution has been improved (1=aggressive search, 0=non-aggressive search, default:0).
- **local_search_type**: Applies a first (=1) or a best (=2) improvement scheme for the local search (Default: 1).
- **decomp**: Decompose the local search (=1) using only the variables perturbed in the global phase. Default: 1.

4.2.3 Output

VNSR output is a list (called **Results** here) containing the following fields:

- **Results$fbest**: Best objective function value found after the optimization.
- **Results$xbest**: Vector providing the best function value found.
- **Results$cpu_time**: CPU Time (in seconds) consumed in the optimization.
- **Results$func**: Vector containing the best objective function value after each improvement.
- **Results$x**: Matrix containing the best vector after each improvement (in rows).
- **Results$time**: Vector containing the CPU time consumed after each improvement.
- **Results$neval**: Vector containing the number of evaluations after each improvement.
- **Results$numeval**: Total number of function evaluations.

The list **Results** as well as **problem** and **opts** are stored and saved in a file called `VNSR_report.RData`.

4.3 Guidelines for using *VNSR*

Parameter tuning in *VNSR* may help increase the efficiency for a particular problem. Here is presented an explanation of the influence of each parameter and suggestions for tuning.

- **opts$use_local**: It is activated (=1) by default but it might be deactivated for a first quick run in which a feasible or a good solution is required in a short computation time, or when the problem dimension is so high that local searches involve high computational times.
- **opts$aggr**: This option uses the aggressive scheme in which the local searh is only applied when the best solution has been improved within the local search. The activation of this options makes the method visit many different neighborhoods but without refining the solutions unless a very good one is found. It might be a good option to locate promising areas or to discard poor areas and constraint the search space later.
- **opts$local_search_type**: There are two types of local searches implemented in the method. One is the first improvement, which perturbs all the decision variables in a random order, changing one unit per dimension and stopping when the intial solution has been outperformed even if there are variables still not perturbed. The second option is a best improvement, which perturbs all the decision variables and then chooses the best solution among the perturbed solution to replace (or not) the initial solution. The best improvement produces a more exhaustive search than the first improvement, but it may be highly time consuming for large-scale problems. The first improvement scheme allows a more dynamic search but can miss refined high quality solutions. In both cases the *go-beyond* principle is applied: If a solution has been improved by perturbing one dimension, we repeat the perturbation following the same direction since it is considered as a promising search direction.

- **opts$decomp**: Performing a local search over all the decision variables might be computationally inefficient in the case of large-scale problems. The Variable Neighbourhood Decomposition Search (VNDS, [?]) decomposes the problem into a smaller sized one by just performing the local search over the perturbed decision variables instead of all of them. The number of perturbed decision variables is determined by the problem dimension and by the options opts$maxdist (see below).
- **opts$maxdist**: This option chooses the percentage (between 0 and 1), of decision variables which are perturbed simultaneously in the search. In other words, it controls the distance between the current solution and the furthest neighborhood to explore. A high percentage (e.g., 100% of the variables) produces a more exhaustive search but is more time consuming. It has been empirically proven that for many instances a low percentage of perturbed variables is efficient to locate global solutions.

4.4 Application example

To illustrate the use of *VNSR* we choose the 10 dimensional Rosenbrock function with integer decision variables as an example. The code of the Rosenbrock function is available inside the installation directory of the *MEIGO* package, under the benchmarks folder. Source the objective function and assign it to the problem list.

```
rosen10<-function(x){
 f<-0;
 n=length(x);
 for (i in 1:(n-1)){
 f <- f + 100*(x[i]^2 - x[i+1])^2 + (x[i]-1)^2;
 }
 return(f)
 }
nvar<-10;
```

Define the problem settings:

```
problem<-list(f="rosen10", x_L=rep(-5,nvar), x_U=rep(1,nvar))
```

Define the algorithm to be used:

```
algorithm="VNS";
```

Define the options for *VNSR*:

```
opts<-list(maxeval=2000, maxtime=3600*69, use_local=1,
 aggr=0, local_search_type=1, decomp=1, maxdist=0.5)
```

Call MEIGO:

```
Results<-MEIGO(problem,opts,algorithm);
```

5 Bayesian inference: BayesFit

5.1 Quick Start

BayesFit is an R implementation of a Bayesian inference method for parameter estimation called BayesSB [?]. It uses MCMC to sample the complete probability distributions of parameters. The calculation steps can be found in Box 1 in [?].

5.2 Quick Start: How to define posterior distributions of parameters with *BayesFit*

We will use a simple logic ODE example [?] where a model of a 3-node network has 6 parameters that need to be optimized to (in-silico generated) time-course data showing the activity of 2 of the nodes.

```
library(MEIGOR)
library(CNORode)
data("simpleExample", package="MEIGOR")
```

The data looks like this:

```
plotCNOlist(cnolist)
```

The model is plotted as follows and a simulation with random values for the parameters can be performed:

```
plotModel(model, cnolist)
initial_pars = createLBodeContPars(model, LB_n=1, LB_k=0.1,
 LB_tau=0.01, UB_n=5, UB_k=0.9, UB_tau=10, random=TRUE)
simData = plotLBodeFitness(cnolist, model, initial_pars,
 reltol=1e-05, atol=1e-03, maxStepSize=0.01)
```

Next, the likelihood functon is defined:

```
f_bayesFit <- function(position, params=initial_pars,
 exp_var=opts$exp_var) {

 # convert from log
 params$parValues = 10^position
 ysim = getLBodeDataSim(cnolist=cnolist, model=model,
 ode_parameters=params)
 data_as_vec = unlist(cnolist$valueSignals)
 sim_as_vec = unlist(ysim)

 # set nan (NAs) to 0
 sim_as_vec[is.na(sim_as_vec)] = 0
 sim_as_vec[is.nan(sim_as_vec)]= 0
 return(sum((data_as_vec-sim_as_vec)^2/(2*exp_var^2)))
 }
```

And the priors for the parameters:

```
prior_mean = log10(initial_pars$parValues)
prior_var = 10
```

The options for the MCMC walk are then defined:

```
opts <- list("model"=NULL, "estimate_params"=NULL,"initial_values"=NULL,
 "tspan"=NULL, "step_fn"=NULL, "likelihood_fn"=NULL,
 "prior_fn"=NULL, "nsteps"=NULL, "use_hessian"=FALSE,
 "rtol"=NULL, "atol"=NULL, "norm_step_size"=0.75,
 "hessian_period"=25000, "hessian_scale"=0.085,
 "sigma_adj_interval"=NULL, "anneal_length"=NULL,
 "T_init"=10, "accept_rate_target"=0.3, "sigma_max"=1,
 "sigma_min"=0.25, "sigma_step"=0.125, "thermo_temp"=1, "seed"=NULL)
opts$nsteps = 2000
opts$likelihood_fn = f_bayesFit
opts$use_hessian = TRUE
opts$hessian_period = opts$nsteps/10
opts$model = list(parameters=list(name=initial_pars$parNames,
 value=initial_pars$parValues))
opts$estimate_params = initial_pars$parValues
opts$exp_var = 0.01
```

And the MCMC walk is then implemented:

```
res = runBayesFit(opts)
```

This function returns the posterior distributions for the parameters as well as other information relating to the MCMC walk. The best-fit parameters can be used to simulate the model and compare to the data:

```
initial_pars$parValues = cur_params(output=res, options=opts)
ysim = plotLBodeFitness(cnolist, model, initial_pars)
```

An application of BayesFit to a larger logic model problem can be found in 7.5.

6 Parallel computation in *MEIGO*

MEIGO allows the user to exploit multi core computation with either of the implemented methods by means of their corresponding parallelizable versions: *CeSSR* and *CeVNS*. The following sections include and introduction to the parallel computing packages needed to exploit this features as well as application examples to implement the methods.

6.1 Quick notes about the parallel computing packages

In order to launch multiple threads of *eSSR* and/or *VNSR* we use the snow package, available in the CRAN repository. Installing and using this package in a cluster might not be entirely trivial and therefore we will start by adding a few notes about this.

6.1.1 Snowfall

The snowfall package is based in the snow package in order to ease cluster programming. Detailed information can be found at http://www.imbi.uni-freiburg.de/parallel/. In *MEIGO* we allow the use of two mechanisms:

- **Socket-connection**: Socket-connection is the simplest option and is what we recommend for a quick-start. Socket connections are made over TCP/IP and do nor require the installation of any additional software. If working in a cluster you will have to specify the address of the machines you want connect to. It might also be worth to take a look at http://www.imbi.uni-freiburg.de/parallel/#passwordless since you will most likely need to configure the ssh-access in order not to be asked for a password each time you initialize a connection.

  If you are running in a local machine (preferentially but no necessarily with multiple cores) the R instances will be launched in your machine. More details on how to configure this options will be provided further ahead in this manual.

- **MPI**: The Message Parsing Interface option depends on your ability to install and use the package Rmpi in your cluster. Detailed information on how to this can be found at http://www.stats.uwo.ca/faculty/yu/Rmpi/.

6.2 Usage

6.2.1 Options

The corresponding cooperative method for either of the algorithms included in *MEIGO* (i.e., *CeSSR* and *CeVNSR*), is invoked using the same problem declaration and options (see sections 3.2 and 4.2). To select the cooperative version of each method, the field `algorithm` must contain either `CeSSR` or `CeVNSR`. The list `opts` must now be a vector for each field instead of a scalar. For example, if we want to use *CeSSR* with 3 different threads, we would define the options of maximum number of evaluations as

```
opts=list();
opts[[1]]$maxeval<-value1
opts[[2]]$maxeval<-value2
opts[[3]]$maxeval<-value3
```

The first n fields should contain the options for the n threads that are going to be used. The field $n+1$ should be named hosts. opts$hosts should be a vector containing as many elements as threads you want to launch in parallel. In case you are using the 'SOCKS' mechanism from snowfall each element of this vector should be a string with the address of the machines where you want to launch the optimization. For instance, opts$hosts('node1','node1','node2','node2','node5','localhost','127.0.0.1') will use 7 threads for each iteration; two at 'node1', two at 'node2', one in 'node5' and two in your local machine ('localhost' and '127.0.0.1').

Additionally, a set of extra options must be defined in the list `opts`. These extra options are the following:

- **ce_maxeval**: The maximum number of evaluations. By default this is set to infinity leaving the stopping criterion to the number of iterations.

- **ce_maxtime**: The maximum time to be spent in the cooperative search. By default this is set to infinity leaving the stopping criterion to the number of cooperative iterations. Notice that although you can specify its value, this stopping criterion will only be checked at the end of each iteration. Therefore if a cooperative iteration takes long it is expected that the cooperative method takes a bit longer to finish than what you have specified.

- **ce_niter**: The number of times the threads will stop to exchange information. The default is 1, meaning that there will be an initial iteration (iteration 0) where the threads will stop to exchange the best solutions found and start a new optimization round. This value can also be set to 0 meaning that you will run multiple *eSSR* or *VNSR* runs in parallel without any exchange of information from the threads.

- **ce_isparallel**: By default this is set to true. Otherwise each thread will be executed sequentially.
- **ce_type**: The type of mechanism used for parallelization. The 'SOCKS' mechanism is incorporated through the snowfall package), In this case, it is important to define the addresses of `opts$hosts` properly. Additionally, the 'MPI' (from 'snowfall') is also available.

6.2.2 Output

The *CeSSR* /*CeVNSR* output is a list containing the following fields:

- **f_mean**: Contains the mean value of the objective function at each iteration.
- **fbest**: Contains the lowest value found by the objective function at each iteration.
- **iteration_res**: Contains the results returned by each *CeSSR* / *VNSR* thread at the end of an iteration. Check sections 3.2.5 and 4.2.3 for more information.
- **numeval**: The number of evaluations at the end of each iteration.
- **time**: The computation time at the end of each iteration.

6.3 *CeSSR* application example

CeSSR is an R implementation of the Cooperative enhanced Scatter Search method which is part of the *MEIGO* toolbox for global optimization in bioinformatics. The *CeSS* strategy [?] is a general purpose technique for global optimization, suited for a computing environment with several processors. Its key feature is the cooperation between the different programs ("threads") that run in parallel in different processors. Each thread implements the enhanced Scatter Search metaheuristic (*eSS*) [?, ?], which is also available in *MEIGO*.

To illustrate the use of *CeSSR* we choose the D/2m-group Shifted and m-rotated Rastrigin's function (f10) from the LSGO benchmarks [?] as an example. The code of f10 is available inside the installation directory of the *MEIGO* package, under the benchmarks folder. Source the objective function and assign it to the problem list, defining the bounds for the decision variables:

```
rosen10<-function(x){
 f<-0;
 n=length(x);
 for (i in 1:(n-1)){
 f <- f + 100*(x[i]^2 - x[i+1])^2 + (x[i]-1)^2;
 }
 return(f)
 }
nvar=20;
problem<-list(f=rosen10, x_L=rep(-1000,nvar), x_U=rep(1000,nvar));
#Set 1 nodes and 2 cpu's per node
n_nodes=1;
n_cpus_per_node=3;
#Set different values for dim_refset, bal and n2
#for each of the 10 cpu's to be used
dim1 = 23;     bal1 = 0;     n2_1 = 0;
dim2 = 33;     bal2 = 0;     n2_2 = 0;
dim3 = 46;     bal3 = 0;     n2_3 = 2;
dim4 = 56;     bal4 = 0;     n2_4 = 4;
dim5 = 72;     bal5 = 0.25;  n2_5 = 7;
dim6 = 72;     bal6 = 0.25;  n2_6 = 10;
```

```
dim7 = 88;      bal7 = 0.25;  n2_7 = 15;
dim8 = 101;     bal8 = 0.5;   n2_8 = 20;
dim9 = 111;     bal9 = 0.25;  n2_9 = 50;
dim10 = 123;    bal10 = 0.25; n2_10 = 100;
opts_dim=c(dim1,dim2,dim3,dim4,dim5,dim6,dim7,dim8,dim9,dim10);
opts_bal=c(bal1,bal2,bal3,bal4,bal5,bal6,bal7,bal8,bal9,bal10);
opts_n2=c(n2_1,n2_2,n2_3,n2_4,n2_5,n2_6,n2_7,n2_8,n2_9,n2_10);
D=10;
#Initialize counter and options
counter=0;
opts=list();
hosts=c();
for(i in 1:n_nodes){
 for(j in 1:n_cpus_per_node){

 counter=counter+1;

 #Set the name of every thread
 if(i<10)hosts=c(hosts,paste('node0',i,sep=""));
 if(i>=10 && i<100)hosts=c(hosts,paste('node',i,sep=""));

 opts[[counter]]=list();

 #Set specific options for each thread
 opts[[counter]]$local_balance          =        opts_bal[counter];
 opts[[counter]]$dim_refset             =         opts_dim[counter];
 opts[[counter]]$local_n2                =        opts_n2[counter];

 #Set common options for each thread

 opts[[counter]]$maxeval                         =        10000;
 opts[[counter]]$local_solver        =        "dhc";

 #Options not set will take default values for every thread

 }
 }
#Set the address of each machine, defined inside the 'for' loop
opts$hosts=c('localhost','localhost','localhost');
#Do not define the additional options for cooperative methods (e.g., ce_maxtime, ce_isparallel, et
#They will take their default values
opts$ce_niter=5;
opts$ce_type="SOCKS";
opts$ce_isparallel=TRUE;
#Call the solver
Results<-MEIGO(problem,opts,algorithm="CeSSR")
```

6.4 *CeVNSR* application example

CeVNSR is an extension of *VNSR* which makes use of parallel computation packages available in R to reduce the time needed to solve a given integer programming problem (IP). This implementation builds on the ideas

explored by [?] which showed (in a nonlinear programming context) that, at least for a set of benchmarks, cooperative instances of an optimization algorithm exchanging information from time to time produced better results than running same instances in an independent fashion with an equivalent computational cost.

Next we show an example function that illustrates how to configure the list of options used by *CeVNSR*. This example calls two cpu's of our local machine to solve the integer Rosenbrock problem. It can be found in the script `main_CeVNSR_example.R` in the benchmarks folder.

```
rosen10<-function(x){
 f<-0;
 n=length(x);
 for (i in 1:(n-1)){
 f <- f + 100*(x[i]^2 - x[i+1])^2 + (x[i]-1)^2;
 }
 return(f)
 }
nvar=20;
problem<-list(f=rosen10, x_L=rep(-1000,nvar), x_U=rep(1000,nvar))
opts=list();
opts[[1]]=list(use_local=1,aggr=1,local_search=1,decomp=1,maxdist=0.8,maxeval=2000);
opts[[2]]=list(use_local=1,aggr=0,local_search=2,decomp=0,maxdist=0.5,maxeval=2000);
opts[[3]]=list(use_local=1,aggr=0,local_search=2,decomp=0,maxdist=0.5,maxeval=2000);
opts[[4]]=list(use_local=1,aggr=0,local_search=2,decomp=0,maxdist=0.5,maxeval=2000);
opts$hosts=c('localhost','localhost','localhost','localhost');
opts$ce_niter=10;
opts$ce_type="SOCKS";
opts$ce_isparallel= TRUE;
Results=MEIGO(problem,opts, algorithm="CeVNSR");
```

7 Applications from Systems Biology

In this section we provide examples for two systems biology problems related with the use and calibration of logic models for modeling cell signaling pathways which motivated the implementation of this toolbox in R.

in [?].

7.1 Using eSS to fit a dynamic model

In this section we use eSS to calibrate a model of ordinary differential equations. Here the dynamics are simulated with the CNORode package [?] which is part of the CellNOptR package [?].

The model shown here is toy pathway for illustrative purposes. A detailed description on the biological problem and published case studies can be found in `http://www.cellnopt.org/`. Briefly, CNORode uses a technique called multivariate polynomial interpolation [?] in order to transform a logic model into a system of ordinary differential equations. The final goal of the package is to calibrate such model (its parameters) by means of optimization strategies where the objective is to minimize the squared difference between the model predictions and the experimental data.

In this example, we start by loading the package and the necessary model and experimental data:

```
library(MEIGOR);
library(CNORode);
data("CNORodeExample",package="MEIGOR");
plotCNOlist(cnolist);
```

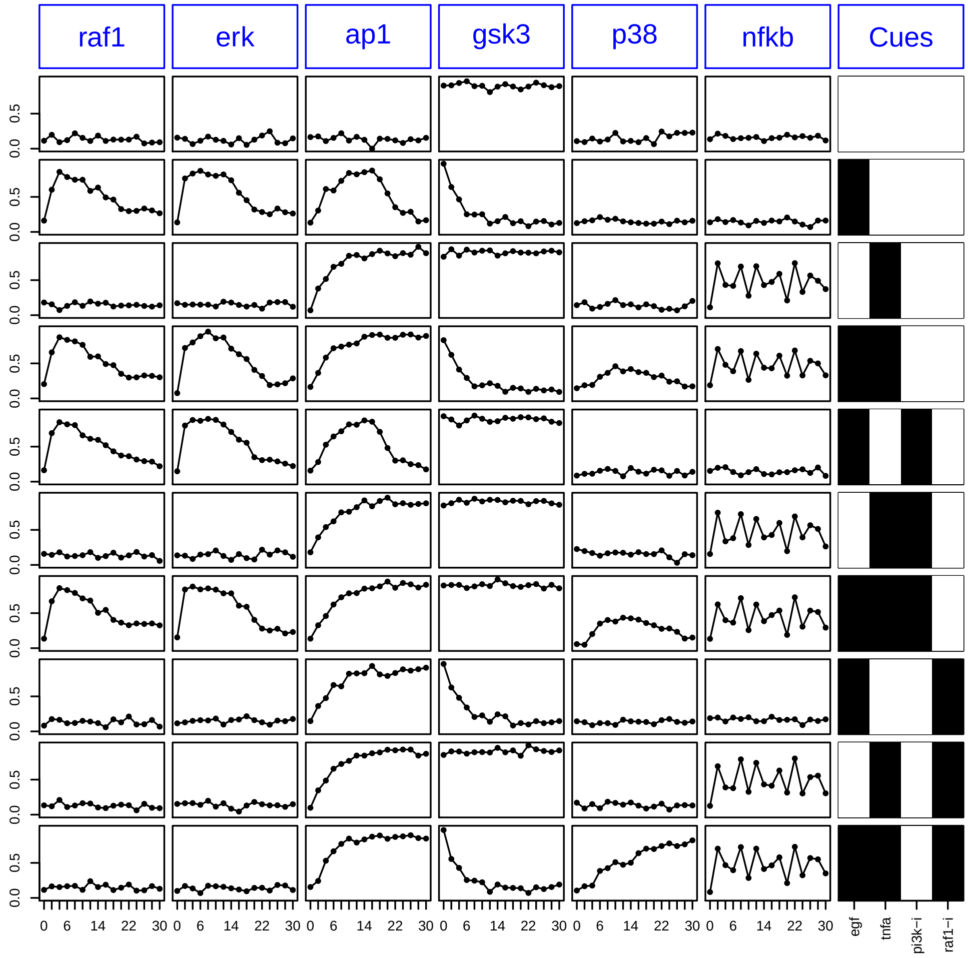

Figure 1: Plot of the experimental data for the CNORode example.

This data was generated *in silico* (*i.e.* by a computer simulation) and added 5% of Gaussian noise to simulate the experimental error. Figure 1 shows the plot of the experimental data.

In order to visualize the logic model we can use the CellNOptR package (see figure 7)

```
plotModel(model, cnolist);
```

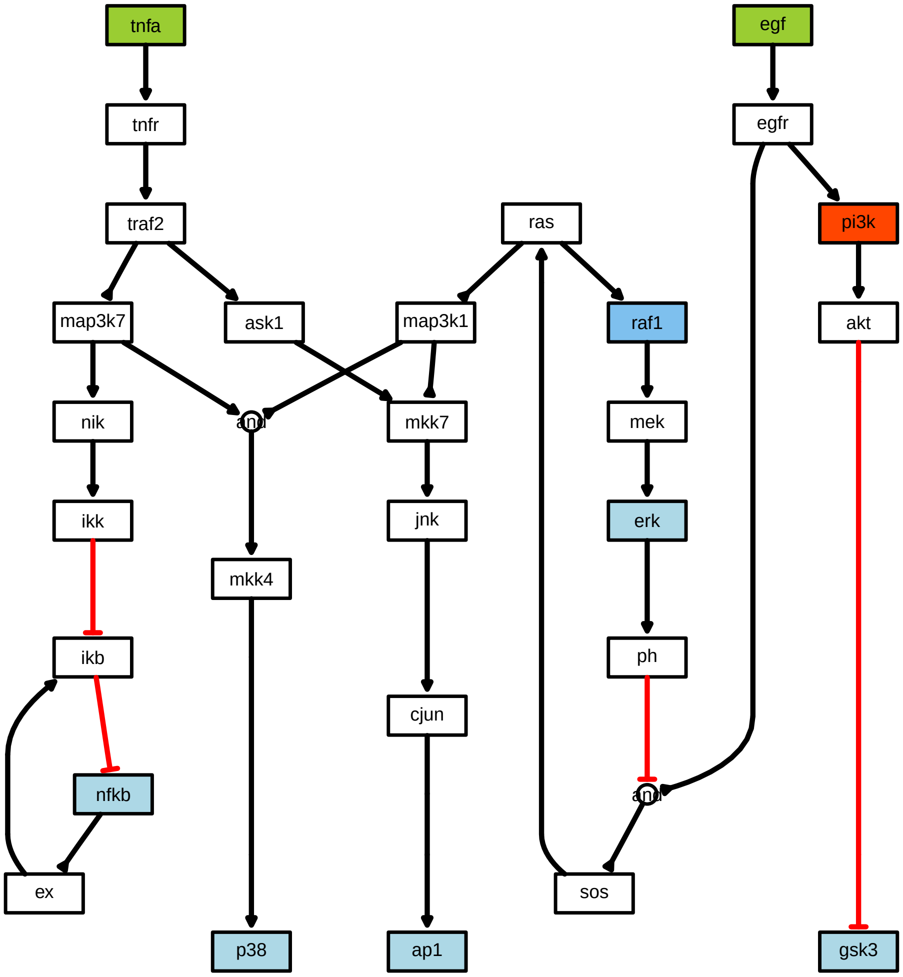

Figure 2: The model structure for the CNORode example.

The models generated by CNORode typically produce a large number of continuous parameters. To automatically generate a list containing the set of continuous parameters and additional information (*e.g.* the parameters names) for this model we can use the *createLBodeContPars* function. Here we set the option *random* to *TRUE* in order to generate a random initial solution for our problem and plot the simulation results against the experimental data (see figure 3).

```
initial_pars=createLBodeContPars(model, LB_n = 1, LB_k = 0.1,
 LB_tau = 0.01, UB_n = 5, UB_k = 0.9, UB_tau = 10, random = TRUE);
opt_pars$parValues=Results$xbest[[3]];
simData=plotLBodeFitness(cnolist, model,opt_pars,reltol = 1e-05, atol = 1e-03,
 maxStepSize = 0.01);
```

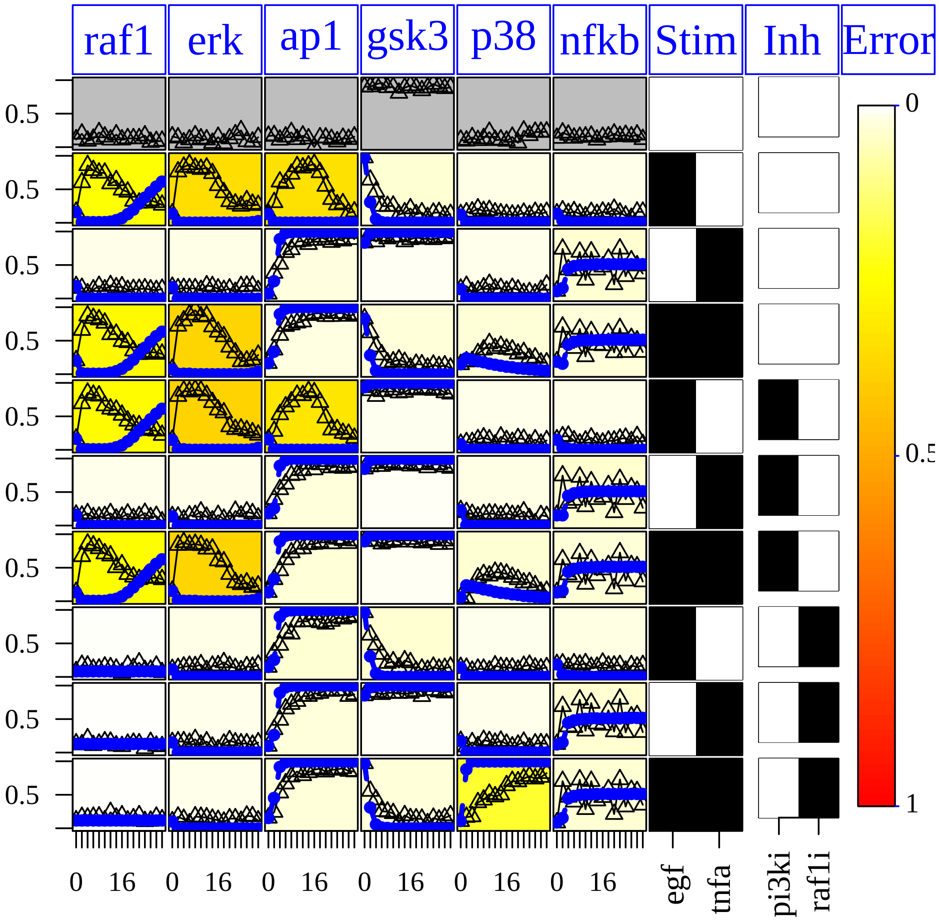

Figure 3: Fit for the initial (random) solution provided to the optimization solver. The simulation is ploted against the experimental data.

The CNORode packages allows the generation of an objective function that can be used by optimization solvers. Basically this function computes the squared difference between the experimental data and the simulation results:

```
f_hepato<-getLBodeContObjFunction(cnolist, model, initial_pars, indices=NULL,
 time = 1, verbose = 0, transfer_function = 2, reltol = 1e-05, atol = 1e-03,
 maxStepSize = 0.01, maxNumSteps = 1e4, maxErrTestsFails = 50, nan_fac = 5)
```

In eSSR an optimization problem is defined by a list that should contain at least the objective function and the upper and lower bound of the variables under study:

```
n_pars=length(initial_pars$LB);
problem<-list(f=f_hepato, x_L=initial_pars$LB[initial_pars$index_opt_pars],
 x_U=initial_pars$UB[initial_pars$index_opt_pars]);
```

Finally the options for the solver must be defined. Please note that the settings used here do not provide the necessary computational effort to solve this problem and are chosen such that the examples can be executed quickly. To improve the performance raise *maxeval*, *ndiverse* and dim_refset and use a local solver (*e.g.* DHC):

```
opts<-list(maxeval=200, local_solver=0,ndiverse=50,dim_refset=10);
```

To start the optimization procedure call MEIGO:

```
Results<-MEIGO(problem,opts,algorithm="ESS")
```

After the optimization we can use the obtained results to plot and plot the model fitness with CNORode (see figure 4):

```
opt_pars=initial_pars;
opt_pars$parValues=Results$xbest;
simData=plotLBodeFitness(cnolist, model,opt_pars,
 reltol = 1e-05, atol = 1e-03, maxStepSize = 0.01);
```

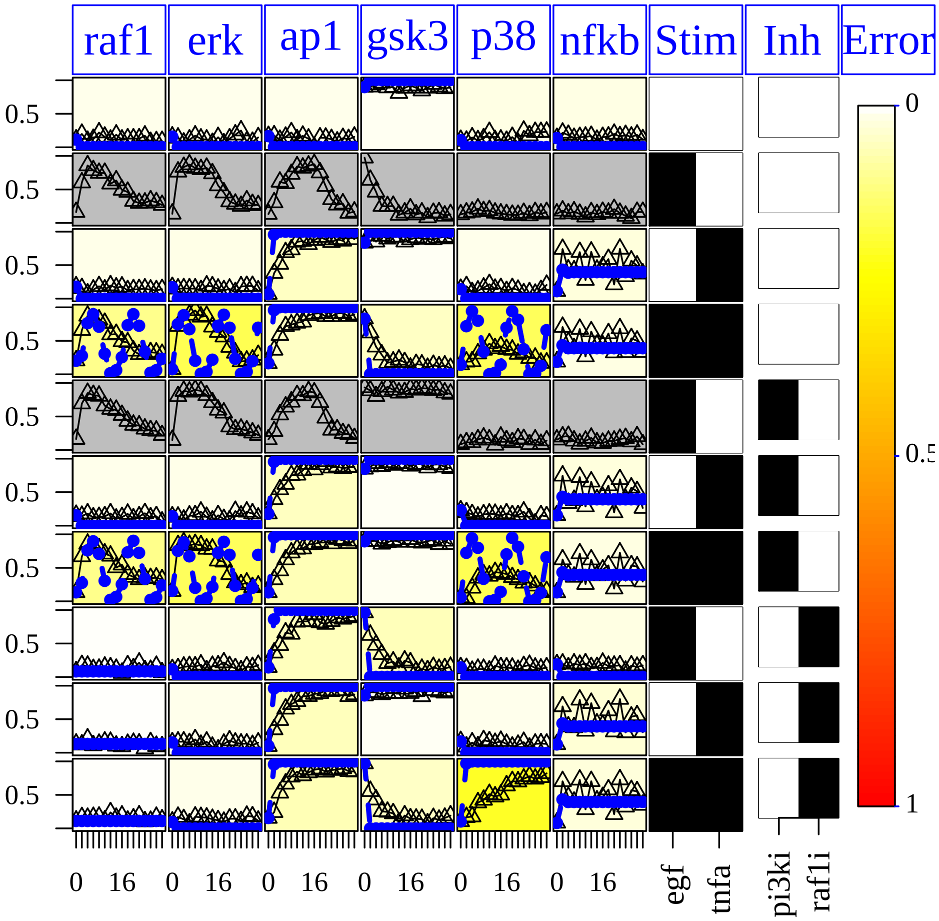

Figure 4: Fitness of the solution obtained by the eSS in the CNORode parameter estimation problem.

7.2 Accelerating the computations with CeSSR

In this section we tackle the same problem described in the previous section but this time using CeSSR in order to accelerate the computations. Here the problem is defined exactly as in the previous case. However, in this case, the options need to be declared as a list, where each element corresponds to the settings for a particular cooperative thread. Additionally, it is essential to specify the host where each thread will be executed:

```
opts=list();
#Set different values for opts
opts[[1]]=list(local_balance=0,dim_refset=10,
 local_n2=1,ndiverse=50,maxeval=200,local_solver=0)
opts[[2]]=list(local_balance = 0.1,dim_refset=11,
 local_n2=2,ndiverse=50,maxeval=200,local_solver=0)
#Set the address of each machine, defined inside the 'for' loop
opts$hosts=c('localhost','localhost');
opts$ce_niter=1;
opts$ce_type="SOCKS";
opts$ce_isparallel=TRUE;
```

The problem can then be solved by calling MEIGO with the algorithm options set as ”CeSSR”:

```
#Call the solver
Results<-MEIGO(problem,opts,algorithm="CeSSR")
```

After the optimization we can use the obtained results to plot the simulation with CNORode (see figure 5):

```
opt_pars=initial_pars;
opt_pars$parValues=Results$xbest[[2]];
simData=plotLBodeFitness(cnolist, model, opt_pars,
 reltol = 1e-05, atol = 1e-03, maxStepSize = 0.01);
```

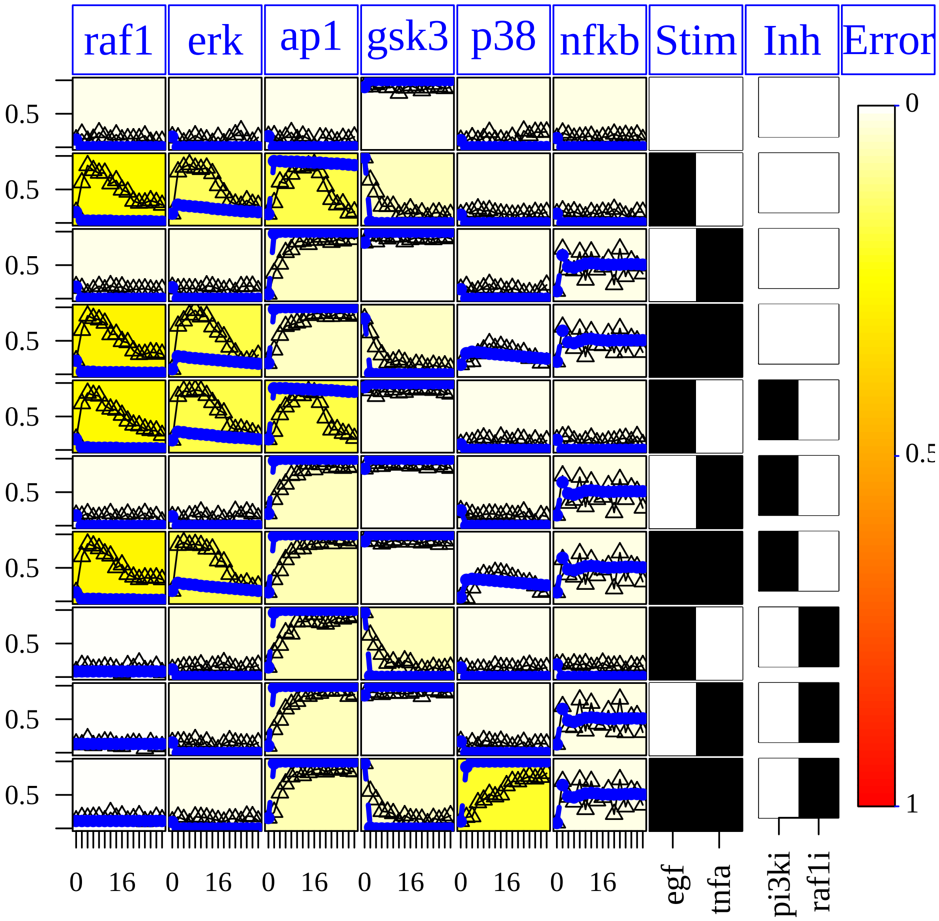

Figure 5: Fitness of the solution obtained by the CeSSR in the CNORode parameter estimation problem.

7.3 Using VNS to optimize logic models

In [?] Saez-Rodriguez and colleagues show how to use a genetic algorithm to optimize a logic model against experimental data. From the optimization point of view, this corresponds to a binary decision problem that can be tackled by VNS.

The basic idea is that each binary decision variable corresponds to a logic disjuntion (an AND gate) from the Boolean point of view or to an hyperedge (an edge with multiple inputs) from the graph perspective. The *CellNOptR* software [?] provides the necessary framework to simulate such models and obtain an objective function that can be used by VNS.

The example provided here is a synthetic pathway, meaning that, both the data and the network structure shown here were engineered for illustrative purposes. Note that a detailed description of the biological problem and published case studies can be found in http://www.cellnopt.org/.

The first step in this example is to load the *CellNOptR* package and a list (the *cnolist*) containing the data for several experiments under different pertubation conditions of upstream network stimuli (typically ligands) and downstream inhibitors (typically small molecule inhibitiors). Also, CellNOptR is used to visualize such data (see figure 6):

```
library(MEIGOR);
library(CellNOptR);
data("CellNOptR_example",package="MEIGOR");
cnolist=CNOlist(cnolist);
plotCNOlist(cnolist)
```

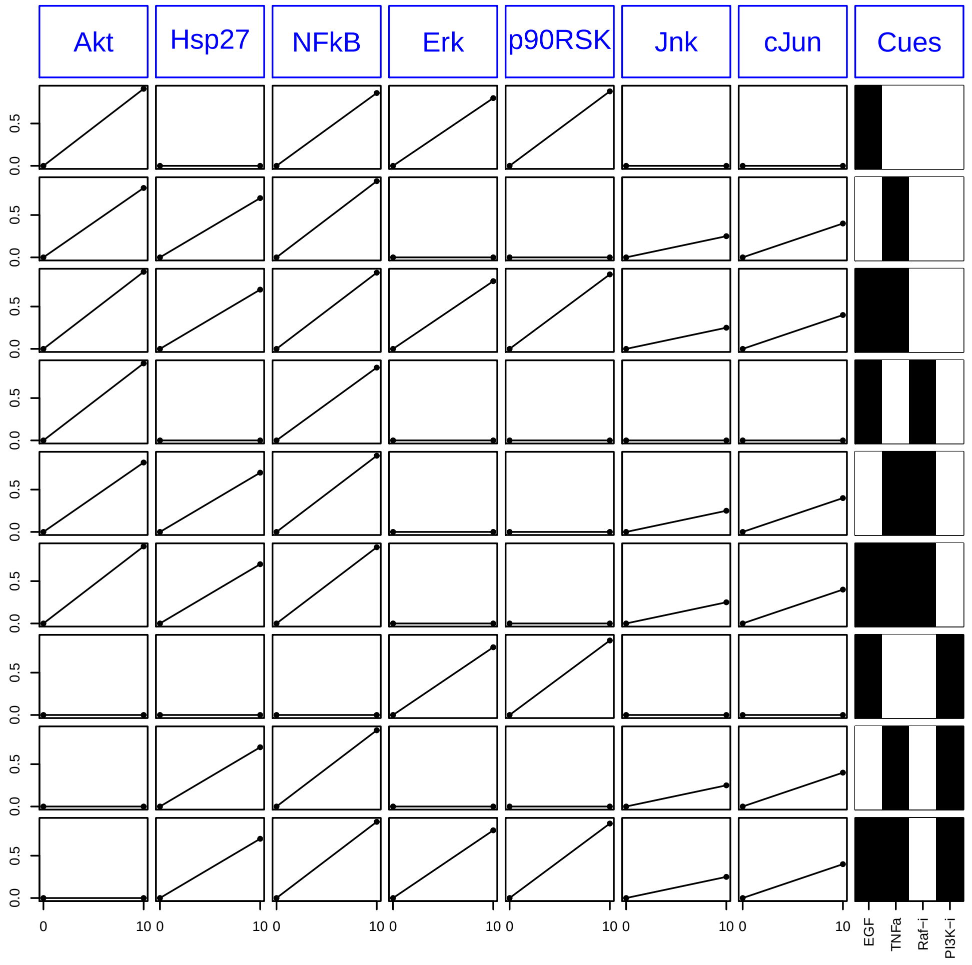

Figure 6: Pseudo-experimental data for the CellNOptR (Boolean logic) example. Each row corresponds to a different experiment upon pertubation with different stimuli and inhibitors.

The original prior-knowledge network (a signed directed graph) can be expanded into an hypergraph containing all possible Boolean gates. CellNOptR can then be used to visualize the obtained network (see figure 7).

```
model <- preprocessing(cnolist, model,
 expansion=TRUE, compression=TRUE, verbose=FALSE);
plotModel(model, cnolist);
```

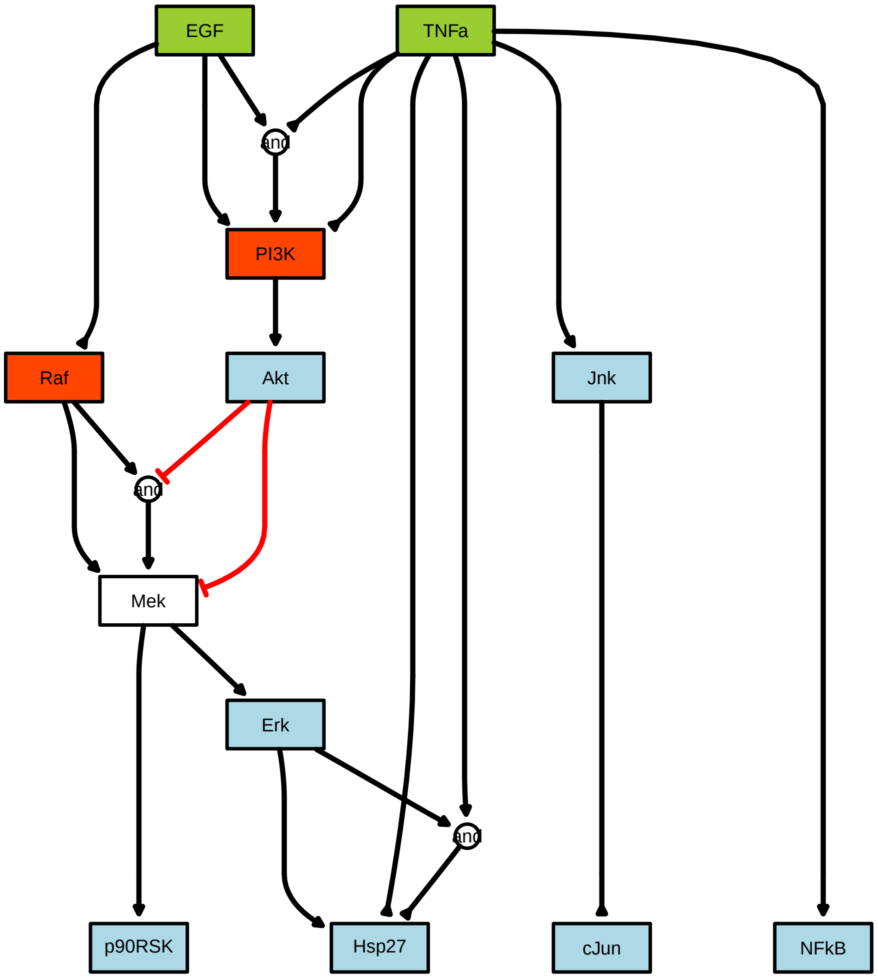

Figure 7: The expanded model for the CellNOptR (Boolean logic) example. This representation contains all possible logic gates where each binary decision variable corrersponds to an hyperedge/logical disjunction.

After loading the experimental data and the prior-knowledge netowrk, we need to define an objective function that can be used by VNS. In order to compute the fitness of the model we will need to perform a simulation and compute the model fitness. Again, CellNOptR automates most of this process and thus our objective function can be defined as follows:

```
get_fobj<- function(cnolist,model){

 f<-function(x,model1=model,cnolist1=cnolist){

 simlist=prep4sim(model1)
 score=computeScoreT1(cnolist1, model1, x)

 return(score)
 }

 }
fobj=get_fobj(cnolist,model)
```

After defining the objective function we need to define the optimization problem and the options for the solver:

```
nvar=16;
problem<-list(f=fobj, x_L=rep(0,nvar), x_U=rep(1,nvar))
opts<-list(maxeval=2000, maxtime=30, use_local=1,
 aggr=0, local_search_type=1, decomp=1, maxdist=0.5)
```

Finally we call MEIGO using the VNS algorithm in order to solve the problem:

```
Results<-MEIGO(problem,opts,"VNS")
```

Once the optimization procedure is finished we plot the obtained solution (see figure 8):

```
optModel=cutModel(model,Results$xbest);
plotModel(optModel,cnolist);
```

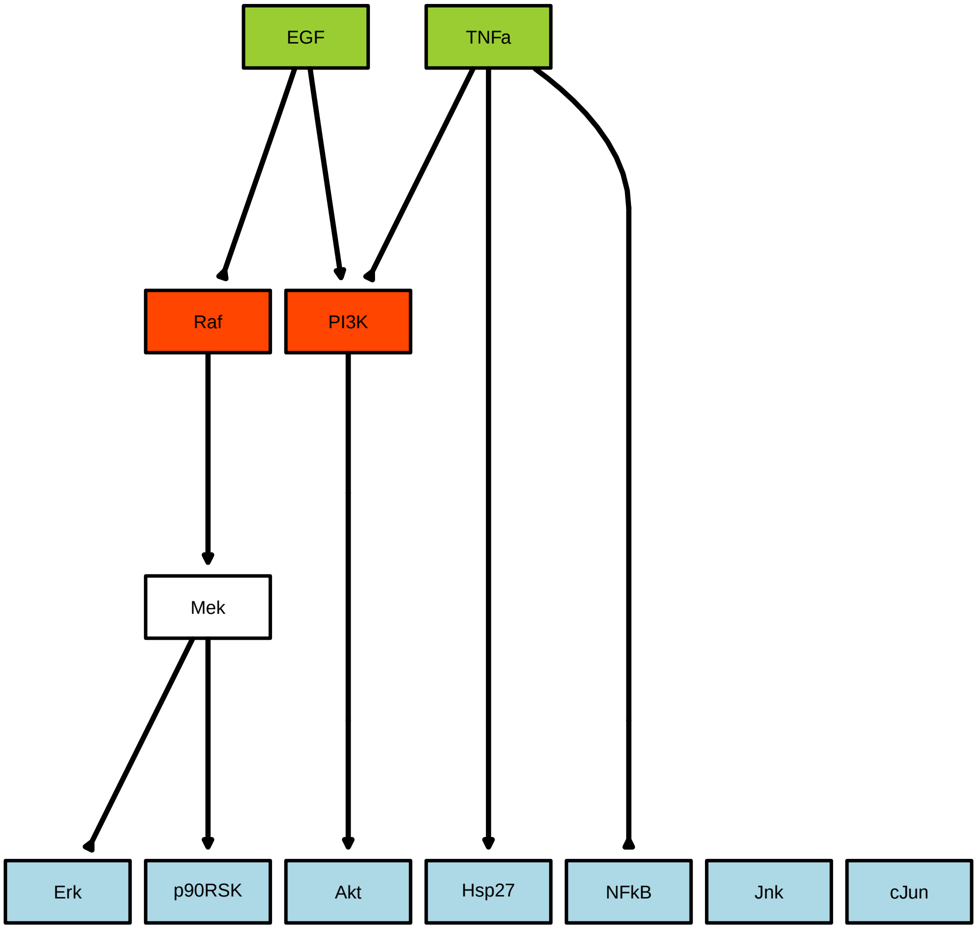

Figure 8: The Boolean logic model obtained after optimization with VNS.

7.4 Using CeVNSR to accelerate computations

In this section we solve the probem of fitting a logic model to experimental data using a cooperative strategy to accelerate VNS computations.

In this case the problem is defined exactly as in the previous section. The main difference with the single thread version described in the previous section is that the option must be defined as a list where each element corresponds to a list containing the options for each cooperative thread:

```
opts=list();
opts[[1]]=list(use_local=1,aggr=1,local_search=1,decomp=1,maxdist=0.8,maxeval=1000);
opts[[2]]=list(use_local=1,aggr=0,local_search=2,decomp=0,maxdist=0.5,maxeval=1000);
opts$hosts=c('localhost','localhost');
opts$ce_niter=2;
opts$ce_type="SOCKS";
opts$ce_isparallel= TRUE;
```

In principle the aim is to diversify these options as much as possible for each thread. The key idea is that in some threads exploration will be favoured against exploitation and the other way around for some other threads. After the problem is properly configured MEIGO can be used to calibrate the logic model using the CeVNSR algorithm.

```
Results=MEIGO(problem,opts,algorithm="CeVNSR",global_save_list=c('model','cnolist'));
```

After the optimization procedure is finished we can use CEllNOptR functionality to plot the obtained model (see figure 9):

```
optModel=cutModel(model,Results$xbest[[3]]);
plotModel(optModel,cnolist);
```

```
optModel=cutModel(model,Results$xbest[[3]]);
plotModel(optModel,cnolist);
```

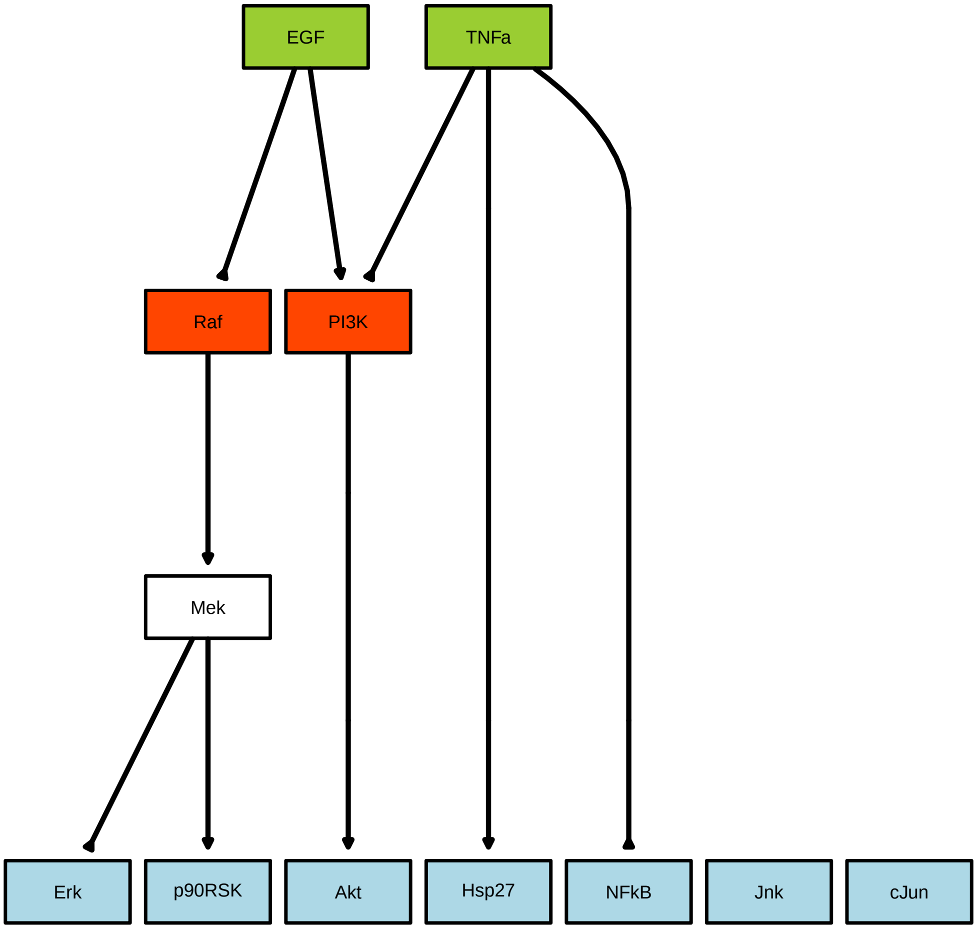

Figure 9: The Boolean logic model obtained after optimization with CeVNSR.

7.5 Using BayesFit to optimize logic models

Here, we can demonstrate the additional information that can be derived from the probability distributions of optimized parameters. The problem is again based on a logic model where, this time, the topology of the model is known and the goal is to optimize the parameters of the transfer functions used to generate a continuous simulation of the model. The parameters were optimized to reduce the distance between the model simulation and in silico generated data. This example is as described in section 6 from [?]; the only difference is that the model used here is the compressed model used to generate the in silico data in [?]. BayesFit produced a good fit to the data (using opts$nsteps=200000), comparable to that of eSS (Mean Squared Error: BayesFit, 0.007; eSS, 0.005).

```
library(MEIGOR)
library(CNORode)
data("bayesFitExample", package="MEIGOR")
```

The data looks like this:

```
plotCNOlist(cnolist)
```

The model is plotted as follows and a simulation with random values for the parameters can be performed:

```
plotModel(model, cnolist)
initial_pars = createLBodeContPars(model, LB_n=1, LB_k=0.1,
 LB_tau=0.01, UB_n=5, UB_k=0.9, UB_tau=10, random=TRUE)
simData = plotLBodeFitness(cnolist, model, initial_pars,
 reltol=1e-05, atol=1e-03, maxStepSize=0.01)
```

Next, the likelihood functon is defined:

```
f_bayesFit <- function(position, params=initial_pars,
 exp_var=opts$exp_var) {

 # convert from log
 params$parValues = 10^position
 ysim = getLBodeDataSim(cnolist=cnolist, model=model,
 ode_parameters=params)
 data_as_vec = unlist(cnolist$valueSignals)
 sim_as_vec = unlist(ysim)

 # set nan (NAs) to 0
 sim_as_vec[is.na(sim_as_vec)] = 0
 sim_as_vec[is.nan(sim_as_vec)]= 0
 return(sum((data_as_vec-sim_as_vec)^2/(2*exp_var^2)))
 }
```

And the priors for the parameters. Here the priors are specified based on the range between the given lower and upper bounds of the parameter types n, k and tau. The range is set to 2 standard deviations. This can be adjusted depending on the preference for narrow or wide priors.

```
prior_width = 2
prior_mean = log10((initial_pars$LB+initial_pars$UB)/2)
prior_var = numeric(length(initial_pars$parNames))
prior_var = (log10(initial_pars$UB-initial_pars$LB)/prior_width)^2
```

The options for the MCMC walk are then defined:

```
opts <- list("model"=NULL, "estimate_params"=NULL,"initial_values"=NULL,
 "tspan"=NULL, "step_fn"=NULL, "likelihood_fn"=NULL,
 "prior_fn"=NULL, "nsteps"=NULL, "use_hessian"=FALSE,
 "rtol"=NULL, "atol"=NULL, "norm_step_size"=0.75,
 "hessian_period"=25000, "hessian_scale"=0.085,
 "sigma_adj_interval"=NULL, "anneal_length"=NULL,
 "T_init"=10, "accept_rate_target"=0.3, "sigma_max"=1,
 "sigma_min"=0.25, "sigma_step"=0.125, "thermo_temp"=1, "seed"=NULL)
opts$nsteps = 2000
opts$likelihood_fn = f_bayesFit
opts$use_hessian = TRUE
opts$hessian_period = opts$nsteps/10
opts$model = list(parameters=list(name=initial_pars$parNames,
 value=initial_pars$parValues))
opts$estimate_params = initial_pars$parValues
opts$exp_var = 0.01
```

And the MCMC walk is then implemented:

```
res = runBayesFit(opts)
```

This function returns the posterior distributions for the parameters as well as other information relating to the MCMC walk. The best-fit parameters can be used to simulate the model and compare to the data:

```
initial_pars$parValues = cur_params(output=res, options=opts)
ysim = plotLBodeFitness(cnolist, model, initial_pars)
```
