# Supplementary material for: MEIGO: an open-source software suite based on metaheuristics for global optimization in systems biology and bioinformatics
Source: BMC Bioinformatics. 2014 May 10;15:136. doi: 10.1186/1471-2105-15-136 (PMC4025564; doi:10.1186/1471-2105-15-136)
Supplement: Additional file 3 — Test of options for the integer programming benchmark problems. The file includes the test carried out with the different search options to solve the integer programming benchmark problems as well as the extracted conclusions. [file 1471-2105-15-136-S3.pdf]

MEIGO: a software suite based on metaheuristics for global optimization in systems biology and bioinformatics

Set of options for solving the integer programming benchmark problems

Different sets of VNS options were tested in order to find the best combination to solve the integer programming benchmark problems. The different sets are showed in the table below.

Options Set	*use_local*	*aggr*	local_search_type	decomp	maxdist
opts01	0	-	-	-	0.8
opts02	0	-	-	-	0.5
opts03	0	-	-	-	0.2
opts04	1	1	0	1	0.8
opts05	1	1	0	1	0.5
opts06	1	1	0	1	0.2
opts07	1	1	0	2	0.8
opts08	1	1	0	2	0.5
opts09	1	1	0	2	0.2
opts10	1	1	1	1	0.8
opts11	1	1	1	1	0.5
opts12	1	1	1	1	0.2
opts13	1	1	1	2	0.8
opts14	1	1	1	2	0.5
opts15	1	1	1	2	0.2
opts16	1	0	0	1	0.8
opts17	1	0	0	1	0.5
opts18	1	0	0	1	0.2
opts19	1	0	0	2	0.8
opts20	1	0	0	2	0.5
opts21	1	0	0	2	0.2
opts22	1	0	1	1	0.8
opts23	1	0	1	1	0.5
opts24	1	0	1	1	0.2
opts25	1	0	1	2	0.8
opts26	1	0	1	2	0.5
opts27	1	0	1	2	0.2

The following tables show the results obtained on each problems using every set of options. Ten runs per options set were carried out (i.e., 270 runs per problem).

As shown in the results, it appears that those runs using local search and $aggr = 0$ are significantly better than the rest. Also, a small percentage of perturbed variables (i.e., $maxdist = 0.2$) shows a poorer performance compared with the results obtained perturbing a higher percentage of variables.

Options Set	Best	Mean		Options Set	Best	Mean
opts01	2.36e-09	8.19e-09		opts15	4.47e-08	1.36e-06
opts02	3.07e-10	1.59e-08		opts16	**2.31e-11**	2.02e-09
opts03	2.36e-09	5.72e-07		opts17	**2.31e-11**	2.02e-09
opts04	1.26e-09	2.85e-08		opts19	1.17e-10	1.58e-09
opts05	1.36e-09	2.83e-08		opts19	**2.31e-11**	1.73e-09
opts06	3.82e-09	1.04e-06		opts20	**2.31e-11**	1.83e-09
opts07	1.36e-09	9.23e-09		opts21	9.92e-10	4.95e-09
opts08	**2.31e-11**	2.66e-08		opts22	8.89e-10	2.05e-09
opts09	2.36e-09	6.95e-07		opts23	9.92e-10	1.14e-08
opts10	1.55e-10	8.78e-09		opts24	2.73e-08	7.27e-07
opts11	**2.31e-11**	8.52e-09		opts25	**2.70e-12**	2.05e-09
opts12	4.47e-08	1.36e-06		opts26	9.92e-10	9.48e-09
opts13	9.92e-10	2.82e-08		opts27	2.73e-08	7.27e-07
opts14	**2.31e-11**	7.78e-09				

Results for *geartrain* problem (1 s. per run)

Options Set	Best	Mean		Options Set	Best	Mean
opts01	13	13		opts15	13	13
opts02	13	13		opts16	13	13
opts03	13	[a]		opts17	13	13
opts04	13	13		opts19	13	13
opts05	13	13		opts19	13	13
opts06	13	[a]		opts20	13	13
opts07	13	13		opts21	13	13
opts08	13	13		opts22	13	13
opts09	13	[a]		opts23	13	13
opts10	13	13		opts24	13	13
opts11	13	13		opts25	13	13
opts12	13	13		opts26	13	13
opts13	13	13		opts27	13	13
opts14	13	13				

[a]One run out of ten did not provide any feasible solution.

Results for *mittelman* problem (1 s. per run)

Options Set	Best	Mean		Options Set	Best	Mean
opts01	5.3	5.3		opts15	5.3	[b]
opts02	5.3	5.3		opts16	5.3	5.3
opts03	5.3	[a]		opts17	5.3	5.3
opts04	5.3	5.3		opts19	5.3	5.3
opts05	5.3	5.3		opts19	5.3	5.3
opts06	5.3	[b]		opts20	5.3	5.3
opts07	5.3	5.3		opts21	5.3	5.3
opts08	5.3	5.3		opts22	5.3	5.3
opts09	5.3	[a]		opts23	5.3	5.3
opts10	5.3	5.3		opts24	5.3	[b]
opts11	5.3	5.3		opts25	5.3	5.3
opts12	5.3	[b]		opts26	5.3	5.3
opts13	5.3	5.3		opts27	5.3	[b]
opts14	5.3	5.3				

[a]Eight runs out of ten did not provide any feasible solution.

[b]Nine runs out of ten did not provide any feasible solution.

Results for *trimlon2* problem (1 s. per run)

Options Set	Best	Mean		Options Set	Best	Mean
opts01	11	12.99		opts15	9.5	13.83
opts02	12	15.14		opts16	8.3	9.3
opts03	9.7	11.53		opts17	8.3	8.88
opts04	12.7	14.44		opts19	8.4	9.16
opts05	10.6	15		opts19	8.4	9.15
opts06	9.5	13.34		opts20	8.4	9.08
opts07	10.6	13.63		opts21	8.5	9.57
opts08	11.7	13.96		opts22	8.3	9.4
opts09	9.5	12.22		opts23	8.3	9.23
opts10	9.9	13.76		opts24	9.5	10.77
opts11	10.6	13.96		opts25	8.3	9.29
opts12	9.5	13.83		opts26	8.3	9.91
opts13	9.8	13.78		opts27	9.4	10.44
opts14	9.7	13.38				

Results for *trimlon4* problem (4 s. per run)

Options Set	Best	Mean		Options Set	Best	Mean
opts01	13.5	18.67		opts15	11.5	18.56
opts02	12.5	16		opts16	10.6	11.53
opts03	12.1	14.64		opts17	11	11.39
opts04	12.2	16.56		opts19	10.8	11.22
opts05	12	[a]		opts19	10.8	11.5
opts06	12.8	16.43		opts20	10.8	11.49
opts07	12.2	17.21		opts21	10.8	11.09
opts08	13.1	18.35		opts22	11	11.52
opts09	13.8	17.3		opts23	10.6	11.47
opts10	12.3	18.21		opts24	10.6	11.99
opts11	12.3	17.88		opts25	10.9	11.46
opts12	11.2	18.2		opts26	10.7	11.68
opts13	13.1	20.24		opts27	10.9	11.82
opts14	14.3	20.4				

[a] One run out of ten did not provide any feasible solution.

Results for *trimlon5* problem (10 s. per run)
