# Supplementary figures and images for: MEIGO: an open-source software suite based on metaheuristics for global optimization in systems biology and bioinformatics
Source: BMC Bioinformatics. 2014 May 10;15:136. doi: 10.1186/1471-2105-15-136 (PMC4025564; doi:10.1186/1471-2105-15-136)

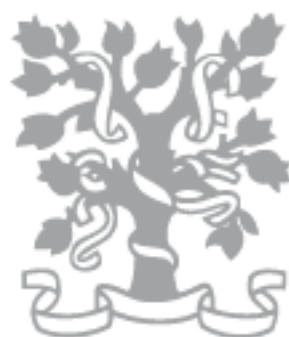

CSIC

CONSEJO SUPERIOR DE INVESTIGACIONES CIENTÍFICAS

Supplement: Additional file 5 — MEIGO R version source code and examples. The file includes the source code of the R version of MEIGO and the examples included in the users manual. [file 1471-2105-15-136-S5.zip › MEIGOR/inst/doc/logo_CSIC-eps-converted-to.pdf]

EMBL-EBI

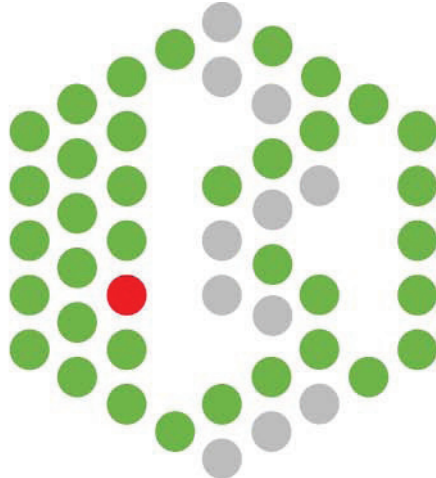

Supplement: Additional file 5 — MEIGO R version source code and examples. The file includes the source code of the R version of MEIGO and the examples included in the users manual. [file 1471-2105-15-136-S5.zip › MEIGOR/inst/doc/logo_EBI-eps-converted-to.pdf]

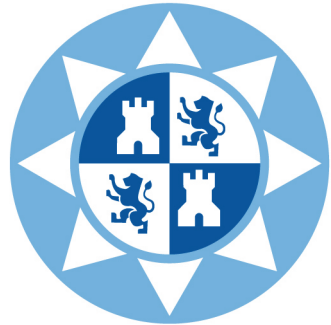

Universidad  
Politécnica  
de Cartagena

Supplement: Additional file 5 — MEIGO R version source code and examples. The file includes the source code of the R version of MEIGO and the examples included in the users manual. [file 1471-2105-15-136-S5.zip › MEIGOR/inst/doc/logo_UPCT-eps-converted-to.pdf]
